# NPWT resource use compared with standard moist wound care in diabetic foot wounds: DiaFu randomized clinical trial results

**DOI:** 10.1186/s13047-022-00569-w

**Published:** 2022-09-30

**Authors:** Dörthe Seidel, Rolf Lefering, DiaFu study group, Martin Storck, Holger Lawall, Gernold Wozniak, Peter Mauckner, Dirk Hochlenert, Walter Wetzel-Roth, Klemens Sondern, Matthias Hahn, Gerhard Rothenaicher, Thomas Krönert, Karl Zink

**Affiliations:** Institut für Forschung in der Operativen Medizin (IFOM), University of Witten/Herdecke, Ostmerheimerstraße 200 Haus 38, 51109 Köln, Germany

**Keywords:** Negative pressure wound therapy, Diabetic foot, Wound care, Wound, Resource use

## Abstract

**Background:**

Diabetic foot ulcers not only have a negative impact on patient mortality, morbidity and quality of life, but also require high resource utilization to achieve complete wound healing. The aim of this evaluation was to compare resource utilization of negative pressure wound therapy (NPWT) and standard moist wound care (SMWC) for diabetic foot wounds after amputation, surgical debridement or wound cleansing.

**Methods:**

The multicenter clinical DiaFu study enrolled 368 adults with diabetic foot ulcers between December 23, 2011 and October 21, 2014. Patients were randomly assigned to NPWT and SMWC. Evaluation of direct resource use comprised inpatient and outpatient treatment time, and personnel and material for wound treatment within 16 weeks. This resource use analysis was primarily based on the per protocol population (NPWT 44; SMWC 110).

**Results:**

Treatment duration was 16 days shorter with NPWT (mean (SD) 82.8 (31.6), SMWC 98.8 (24.6); U test, *p* = 0.001) with 14.9 days shorter outpatient treatment (mean (SD) NPWT 68.3 (31.1), SMWC 83.2 (29.7)). The number of dressing changes per study participant was lower with NPWT (mean (SD) 35.1 (18.6), SMWC (42.9 (21.4); U test, *p* = 0.067). Time per dressing change was significantly lower with SMWC (mean (SD) 19.7 (12.8), NPWT (16.5 (8.2) minutes; U test, *p* < < 0.0001). Time for surgical debridements per study participant was 23.3 minutes shorter with NPWT (mean (SD) 20.5 (20.5), SMWC (43.8 (46.7); U test, *p* = 0.395).

**Conclusions:**

Resource use was lower for NPWT, which may be an efficient treatment alternative to SMWC for diabetic foot wounds, to be demonstrated in subsequent cost analyses.

**Trial registration:**

clinicaltrials.govNCT01480362 on November 28, 2011

**Supplementary Information:**

The online version contains supplementary material available at 10.1186/s13047-022-00569-w.

## Background

Diabetic foot ulcers are common, complex, and costly complications of diabetes [1–3]. Wound complications not only affect patients’ health status, quality of life, and likelihood of survival, but are also associated with a negative economic impact [3]. The type of wound treatment, particularly surgical treatment, was identified as the strongest predictor of high resource use [4]. A significant number of resources for chronic wound care are used in outpatient settings and in home care [5].

Negative pressure wound therapy (NPWT) was introduced into clinical practice in the early 1990s [6–8] and became a widely used wound treatment method considered to be a potentially effective alternative to standard moist wound care (SMWC) [9–13]. Furthermore, published literature examining the use of NPWT in wounds of various origin demonstrated that NPWT could reduce rehospitalizations, associated surgical procedures, dressing changes, personnel commitments, hospitalization and treatment time, time until subsequent surgery and cost [14–18]. For diabetic foot ulcers, Armstrong et al. reported lower average total costs per participant with NPWT than with moist dressings [19]. A subsequent resource utilization analysis showed that there was no difference between groups for in-patient hospital stay (number of admissions or length of stay), significantly more surgical procedures (including debridement), dressing changes and outpatient treatment visits were required in the moist wound treatment group at higher cost [16]. Vaidhya et al. reported significantly lower mean number of dressings applied to achieve satisfactory healing and lower average costs with NPWT using a modified KCI V.A.C. therapy kit [20]. Two analyses based on economic models concluded that, compared to patients treated with advanced wound care, patients treated with NPWT had increased quality-adjusted life years and a higher healing rate at a lower cost [15, 21]. However, there is still an increasing demand for quality outcome data to support the economic decision-making process with attention to resource use efficiency and assessment of consequence rather than simplistic cost arguments [22].

For diabetic foot wounds treated with NPWT compared to SMWC in the DiaFu study, we previously reported no significant difference of wound closure rate in the intention-to-treat (ITT) and in the per protocol (PP) population [23]. Time to wound closure was significantly shorter with NPWT in the PP population, but there was no significant difference between the treatment arms in the ITT population. A significantly higher number of study participants with NPWT had at least one adverse event but the number of events related to NPWT were low. The validity of the ITT population results was weakened by protocol violations and missing and incomplete endpoint documentation.

The purpose of this evaluation was to compare inpatient and outpatient resource use for wound treatment with NPWT and SMWC for diabetic foot wounds and to provide the results of a complementary analysis for wound closure rate and time using all documented wound closures, for which there was no evidence of reopening within 14 days after initial wound closure to support subsequent cost analyses.

## Methods

### Study design

This resource use evaluation was performed as part of the randomized clinical DiaFu trial which was conducted in 40 hospitals and outpatient facilities in Germany. The study was registered with the ClinicalTrials.gov Identifier: NCT01480362. The study protocol and the informed consent documents were approved by the lead ethical committee of the Witten/Herdecke University.

### Participants

Patients with at least 4-week-old chronic diabetic foot ulcers according to Wagner 2-4 were enrolled in the study after providing written informed consent. The originally planned minimum ulcer age of 6 weeks was reduced to 4 weeks during the course of the study. Patients were excluded if any of the following criteria were met: Age < 18 years, pregnancy, existing or anticipated noncompliance with study requirements as assessed by the local investigator at the time of enrollment, indication for amputation above the lower ankle of the same extremity, necrotic tissue with existing eschar that could not be debrided, untreated osteitis or osteomyelitis, unexplored fistula, malignancy of the wound, exposed nerves, vessels, or anastomotic areas, intended or necessary outpatient NPWT in patients with anticoagulation or more severely impaired coagulation function and increased risk of bleeding with relevant circulatory effects, allergy to a component of the respective treatment arm, severe anemia that was not due to infection, concurrent participation in other interventional studies/previous participation in this study. Because public health insurers offered integrated care contracts for outpatient NPWT, only patients who were members of a participating health insurer were enrolled.

### Randomization and masking

Patients were randomly allocated to the treatment arms in a 1:1 ratio using a computer-generated list located on a centralized web- based tool. Patients were stratified by study site and by Wagner-Armstrong stage within each site (<Wagner-Armstrong stage 2C and ≥ Wagner-Armstrong stage 2C). The randomization lists were generated with the help of a self-created Java program and integrated into the study database. Each registered investigator received individual access to the randomization tool via the study website but without knowledge of future treatment assignment, which provided adequate allocation concealment. The investigators were responsible for adequately implementing the assigned treatment. Due to the physical differences between the treatment regimens, it was not possible to blind either participant or physician to the treatment assignment. Verification of complete wound closure was performed by independent, blinded assessment of wound photographs. Determination of wound size and percentage wound tissue quality was also performed by central, blinded outcome assessors based on the wound photographs using the Wound Healing Analyzing Tool (W.H.A.T.). The determination of sufficient wound bed conditioning and the indication for surgical procedures was carried out by the treating physician, as in clinical practice.

### Procedures

Prior to randomization and initiation of study treatment, patients received either a surgical debridement, an amputation of foot parts, or a thorough wound cleansing, depending on the individual wound needs, to allow clear assessment of the wound and to provide optimal conditions for study treatment with the aim of complete wound healing. Amputations involved part or the entire study wound, changing the chronic state to an acute condition.

At baseline, patients received an extensive examination of the study wound, actual surgical history, and overall health status. Study treatment started either outpatient or in-hospital and was to be continued in outpatient care whenever possible. Diagnosis and therapy of the causative diseases were performed according to national guidelines (German National Health Care Guideline Type 2 Diabetes (available in various versions at https://www.awmf.org/leitlinien) and Type 2 Diabetes - Prevention and Treatment Strategies for Foot Complications (available in the archive at https://www.leitlinien.de/) as part of routine clinical practice at the discretion of the treating physician.

In the intervention arm, commercially available CE-marked NPWT devices of the participating manufacturers (Kinetic Concepts Incorporated (KCI), an Acelity company, now part of 3 M and Smith & Nephew (S&N)) were used in the discretion of the clinical investigator in compliance with manufacturers’ recommendations for use (available on the manufacturers’ websites). Intermittent and continuous NPWT was allowed to be used with the negative pressure to be adapted as recommended for the dressing applied and adapted to the wound needs. NPWT as interim therapy was discontinued once the condition of a wound was suitable either for surgical closing or for healing by epithelialization.

Control therapy was SMWC, which was any local wound treatment regularly used in the respective study site that did not have an experimental status or was NPWT. SMWC was applied according to the hospitals’ local clinical standards and guidelines, based on the individual needs of the wound in the process of healing, and with special attention paid to exudate amount and local infection status.

In both treatment arms, wound-related procedures were performed when considered clinically necessary. Wounds were closed either surgically or healed by secondary intention. In the NPWT arm, secondary healing was achieved with SMWC dressings after NPWT was discontinued.

Maximum study treatment time was 16 weeks after randomization. Study visits were performed after week 1, 3, 5, 12 and 16, and in the event of end of treatment, hospital discharge, wound closure and for wound closure confirmation after a minimum of 14 days. The observation period of 16 weeks was chosen based on the experience of clinical experts and the results of the study by Blume et al. [24] assuming that the majority of wounds can be closed within this period. Study participants were followed up until 6 months after randomization. The initially planned follow-up period of 12 months was reduced to 6 months in the course of the study.

Data were collected using electronic case report forms. Quality assurance of data collection was ensured by 100% monitoring of demographic data, selected baseline data, and primary endpoint data. Random monitoring was performed for all other end points, with the number of controls adjusted according to data quality. Clinical research associates visited study sites at least three times during the study (initiation, data monitoring, completion), with the frequency of visits adjusted for the number of randomized patients per study site.

### Outcomes

The resource assessment included parameters for direct wound care with dressings and related support activities like wound cleansing, surgical debridement, wound edge and wound environment protection, pressure relief, revascularization, amputations and coverage of tissue defects. The evaluation comprised type and extent of interventions performed and if applicable, time, personnel and material used. Time recording included inpatient and outpatient treatment length, length of hospital-stay, time to hospital discharge, (re) hospitalizations, and time spent on wound treatment and supportive care. Personnel recording included the activities of assistant and specialist physicians, nurses, nursing assistance and other personnel (e.g. podiatrists). Material recording comprised dressings and systems used for NPWT and the categories of wound dressings (primary (filler) and secondary (cover) dressing; see also Table 6) used for SMWC. The time per dressing change and the personnel required were determined by means of exemplary surveys during each visit in the respective study site.

In addition to the analysis of clinical effectiveness, the number of closed wounds within 16 weeks for which there was no evidence that they were not closed or reopened within 14 days was determined. In this context, wound closures were accepted where wound photographs were missing or of poor quality, and study visits to confirm wound closure after 14 days were missing or documentation was missing.

We previously published the time to achieve optimal preparation of the wound for further therapeutic measures at 95% granulation of the wound bed based on the data for the first achievement of this direct therapeutic goal within 16 weeks [23]. However, a closer look at the treatment courses in the present resource utilization analysis showed that due to events (e.g., surgical interventions), the last time point of reaching 95% granulation within the study treatment period had to be used. Adjusted results for this endpoint are presented. In addition, information on continuation of treatment after 95% granulation of the study wound was achieved has been added.

### Statistical analysis

Sample size calculation was performed using the expected difference between wound closure rates in both treatment arms based on information extracted from previously published studies by Armstrong and Lavery 2005 [19] and Blume et al. 2008 [24]. We assumed a complete wound closure rate of 45% for NPWT and 30% for SMWC, resulting in a minimum difference of 15% after a treatment time of 16 weeks. Based on a type one error of α = 0.05 and a type 2 error of β = 0.2 (corresponding to a power of 80%), a total sample size of 162 patients per group was calculated. The computer program of Dupont and Plummer was used for sample size calculation [25].

**Fig. 1:**
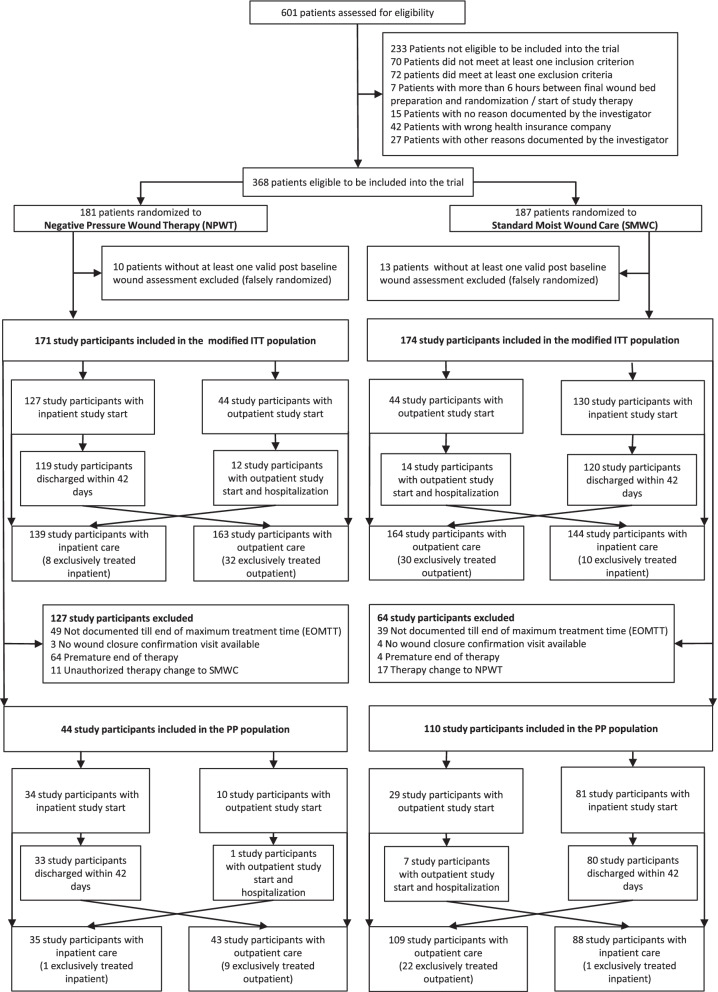
Study Participant Flow Diagram in the Diabetic Foot (German: Diabetischer Fuß) (DiaFu) Randomized Clinical Trial. Patient flow diagram according to Consolidated Standards of Reporting Trials (CONSORT), including reasons for exclusions from the per protocol (PP) population and distribution of study participants across treatment sectors (inpatient and outpatient care). SMWC, conventional wound treatment; EOMTT, end of maximum treatment time; NPWT, negative pressure wound therapy; WC, wound closure; WCC, wound closure confirmation

Resource use analysis was primarily based on the PP population including study participants with complete documentation until the end of the maximum study treatment duration of 16 weeks or complete and verified wound closure was achieved and sustained wound closure was confirmed after 14 days, without premature end of therapy and without change of treatment without reason. However, the results of the modified ITT population including all randomized participants with a valid baseline and at least 1 post baseline wound assessment (Fig. 1) were analyzed secondarily, since this study population corresponds to real-life.

Resource use parameters are presented descriptively with number and percentage (%), mean and standard deviation (SD) and / or 95% confidence interval (CI), minimum (Min) and maximum (Max) per study participant or per treatment procedure as applicable. The unit of time is minutes. Statistical significance was determined using the Chi-squared or Mann Whitney U test, respectively, with an alpha level of 0.05. SPSS statistical software, version 23 (IBM Inc., Armonk, New York), was used for all analyses. If a statistical test was not indicated, this was marked with not applicable (NA).

## Results

Between December 23, 2011 and October 21, 2014, 368 patients were randomized in 40 study sites with the last patient follow-up visit on February 12, 2015. Recruitment was completed. 154 study participants were analyzed in the PP population. The patient flow according to Consolidated Standards of Reporting Trials (CONSORT), including inpatient and outpatient care periods is provided in Fig. 1. The study protocol was published [26]. Reasons for screening failures and exclusion from the ITT and the PP population were previously reported [23].

### Demographics and baseline characteristics

Demographics, wound size and information on wound preparation before treatment start of the PP population are provided in Table 1.

**Table 1:** Demographics, wound size, revascularization and wound surgery before study start in the PP population

Baseline parameter of the PP population	NPWT	SMWC
**Study participants in the population, No.**	**44**	**110**
**Age in years,** Mean (SD)	66.5 (11.0)	67.8 (10.4)
**Sex,** No. (%)		
Male	29 (65.9)	84 of 110 (76.4)
Female	15 (34.1)	26 of 110 (23.6)
**Wound surface area at randomization calculated from CRF entries (width and length),** mm^2^		
Study participants with data available (used from screening), No.	43 (1)	110 (0)
Mean (SD)	964 (1392)	878 (1266)
Min-Max	20 - 7536	12 - 7654
**Wound volume at randomization calculated from CRF entries (width, length and depth),** mm^3^		
Study participants with data available (used from screening), No.	43 (1)	110 (0)
Mean (SD)	33,359 (95748)	14,742 (36523)
Min-Max	94 - 602,880	0 - 306,150
**Study participants with revascularization before study start, No. (%)**	1 (2.3)	8 (7.3)
Study participants with Percutaneous Transluminal Angioplasty (PTA), No. (%)	0 of 1 (0)	5 of 8 (62.5)
Study participants with PTA and Stent, No. (%)	0 of 1 (0)	0 of 8 (0)
Study participants with Venous Bypass, No. (%)	1 of 1 (100)	0 of 8 (0)
Study participants with Polytetrafluoroethylene Bypass, No. (%)	0 of 1 (0)	1 of 8 (12.5)
Study participants with Thromboendarterectomy, No. (%)	0 of 1 (0)	0 of 8 (0)
Study participants with Thromboendarterectomy and Patch plastic, No. (%)	0 of 1 (0)	2 of 8 (25.0)
Study participants with multi-level reconstruction, No. (%)	0 of 1 (0)	0 of 8 (0)
**Study participants with wound surgery at the study wound site before treatment start, No. (%)**	31 (70.5)	83 (75.5)
Study participants with surgical debridement, No. (%)	13 of 31 (41.9)	41 of 83 (49.4)
Study participants with major amputations, No. (%)	0 of 31 (0)	0 of 83 (0)
Study participants with minor amputations, No. (%)	3 of 31 (9.7)	19 of 83 (22.9)
Study participants with minor amputations in the border zone to vital tissues, necrosectomy and debridement, No. (%)	15 of 31 (48.4)	23 of 83 (27.7)

### Inpatient and outpatient treatment periods

In the PP population, treatment began in the hospital for the majority of study participants (NPWT 34 (77.3%), SMWC 81 (73.6%)). In a total of 39 study participants (25.3%), treatment started on an outpatient basis (NPWT 10 (22.7%), SMWC 29 (26.4%)). Nearly all study participants who started treatment as inpatients were discharged during the 16-week treatment period of the study (NPWT 33 of 34 (97.1%), SMWC 80 of 81 (98.8%), *p* = 0.523, Chi-squared test). Time to first discharge was slightly shorter with NPWT (mean difference 1.4 days; U test; *p* = 0.825). For more information on continuation of treatment after hospital discharge and subsequent wound closure, see Table 1 in the Additional file 1.

Length of outpatient care was significantly shorter with NPWT (mean difference 14.9 days; U test; *p* = 0.004) (Table 2). Length of hospital stay was much shorter than the outpatient period and differed little between treatment arms (mean difference 1.1 days; U test; *p* = 0.950) (Table 2). Hospital (re) admission rate was approximately the same in both treatment arms (NPWT 11 of 44 (25.0%), SMWC 27 of 110 (24.5%)).

**Table 2:** Care status and treatment periods during the active study treatment time of 16 weeks in the PP population

Randomized treatments arms / statistical test	NPWT	SMWC	***p*** value (test)
**Study participants in the PP population, No.**	**44**	**110**	**NA**
**Length of care (days)**			
Study participants with data available, No.	44	110	
Mean (SD)	87.1 (31.1)	100.6 (23.4)	0.004 (U)
Min-Max	5 - 112	21 - 112	
**Study participants with inpatient care, No. (%)**	35 (79.5)	88 (80.0)	0.949 (Chi-squared)
**Study participants exclusively treated inpatient, No. (%)**	1 (2.3)	1 (0.9)	0.500 (Chi-squared)
**Length of hospital stay (days)**			
Mean (SD)	14.6 (17.4)	15.7 (21.7)	0.950 (U)
Min-Max	0 – 67	0 – 112	
**Study participants with outpatient care, N (%)**	43 (97.7)	109 (99.1)	0.500 (Chi-squared)
**Study participants exclusively treated outpatient, N (%)**	9 (20.5)	22 (20.0)	0.949 (Chi-squared)
**Length of outpatient care (days)**			
Mean (SD)	68.3 (31.1)	83.2 (29.7)	0.004 (U)
Min-Max			
***Days without treatment***	*189*	*190*	*NA*
**Length of treatment (days)**			
Study participants with data available, No.	44	110	0.001 (U)
Mean (SD)	82.8 (31.6)	98.8 (24.6)	
Min-Max	5 – 112	14 – 112	
**Length of NPWT (days)**			
Study participants with data available, No.	44	0	NA
Mean (SD)	31.2 (32.3)		
Min-Max	1 - 112		
**Length of inpatient NPWT**			
Study participants with data available, No.	35	0	NA
Mean (SD)	10.3 (9.1)	NA	NA
Min-Max	2 – 33		
**Length of outpatient NPWT (days)**			
Study participants with data available, No.	28	0	NA
Mean (SD)	36.1 (33.0)		
Min-Max	1 – 112		
**Length of SMWC (days)**			
Study participants with data available, No.	38	110	NA
Mean (SD)	59.8 (33,4)	98.8 (24.6)	
Min-Max	2 - 110	14 - 112	
**Length of inpatient SMWC (days)**			
Study participants with data available, No.	22	88	
Mean (SD)	13.6 (13,3)	19.6 (22.7)	NA
Min-Max	1 – 55	1 – 112	
**Length of outpatient SMWC (days)**			
Study participants with data available, No.	36 (81.8%)	109 (99.1%)	
Mean (SD)	55.8 (33.0)	83.9 (28.7)	NA
Min-Max	2 – 108	10 – 112	
**Study participants still in care at day 112, N (%)**	17 (38.6%)	66 (60.0%)	0.016 (Chi-squared)

Total treatment length within 16 weeks was significantly shorter in the NPWT arm (mean difference 16 days; U test; *p* < 0.001) (Table 2). Only 6 study participants (13.6%) were treated exclusively with NPWT. Both, NPWT and SMWC were predominantly performed on an outpatient basis (Table 2).

Significantly more study participants with NPWT achieved 95% granulation of the study wound within 16 weeks (difference 30.9%; Chi-squared test; *p* < 0.001) (Table 3). After reaching this therapeutic goal the majority of participants continued treatment with SMWC in both treatment arms. Length of SMWC after 95% granulation was only marginally longer in the SMWC arm than in the NPWT arm.

**Table 3:** Time until finally achieving 95% granulation of the study wound and treatment continuation after optimal preparation for further therapeutic measures within 16 weeks in the PP population

Randomized treatments arms / statistical test	NPWT	SMWC	***p*** value (Test)
**Study participants in the PP population, No.**	44	110	NA
**Study participants without achieving 95% granulation of the study wound, No. (%)**	8 (18.2%)	54 (49.1%)	< 0.001 (Chi-squared)
**Study participants with achieving 95% granulation of the study wound, No. (%)**	36 (81.8%)	56 (50.9%)	
**Time until finally achieving 95% granulation of the study wound within 16 weeks**			
Mean (SD)	33.9 (30.6)	62.0 (37.4)	0.003 (U)
[95% CI]	[21.8-46.0]	[50.8-73.3]	
Min-Max	0 – 111	0 - 115	
**Study participants without data after 95% granulation of the study wound, No. (%)**	0 of 36 (0)	15 of 56 (26.8)	NA
**Study participants with data after 95% granulation of the study wound, No. (%)**	36 of 36 (100)	41 of 56 (73.2)	NA
**Study participants with SMWC after 95% granulation of the study wound, No. (%)**	32 of 36 (88.9)	35 of 41 (85.4)	NA
**Length of SMWC treatment after 95% granulation of the study wound**			
Mean (SD)	61.3 (32.5)	65.5 (36.2)	0.411 (U)
Min-Max	14 – 107	1 – 112	
**Length of inpatient SMWC treatment after 95% granulation of the study wound**			
Study participants with data available, No.	13	17	
Mean (SD)	19.2 (13.9)	12.7 (9.1)	0.145 (U)
Min-Max	6 – 56	1 – 35	
**Length of outpatient SMWC treatment after 95% granulation of the study wound**			
Study participants with data available, No.	31	34	
Mean (SD)	55.8 (32.7)	61.6 (33.9)	0.412 (U)
Min-Max	2 – 107	1 – 112	

Significantly more study participants with NPWT were still in care at the end of week 16 (difference 21.4%; Chi-squared test; *p* = 0.016) (Table 2), with the vast majority of study participants being treated as outpatients at this time (NPWT 17 (100%), SMWC 63 (95.5%)). Only 3 (4.5%) participants in the SMWC arm were hospitalized. At the end of the maximum treatment period, study participants in both the NPWT arm (11 (64.7%)) and the SMWC arm (64 (97.0%)) received predominantly SMWC. Only 4 participants (23.5%) in the NPWT arm were still with NPWT.

Data on local wound treatment during follow-up are provided in Table 2 in the Additional file 1.

### Post hoc analysis of wound closures without evidence for reopening within 14 days after initial wound closure within 16 weeks

**Fig. 2:**
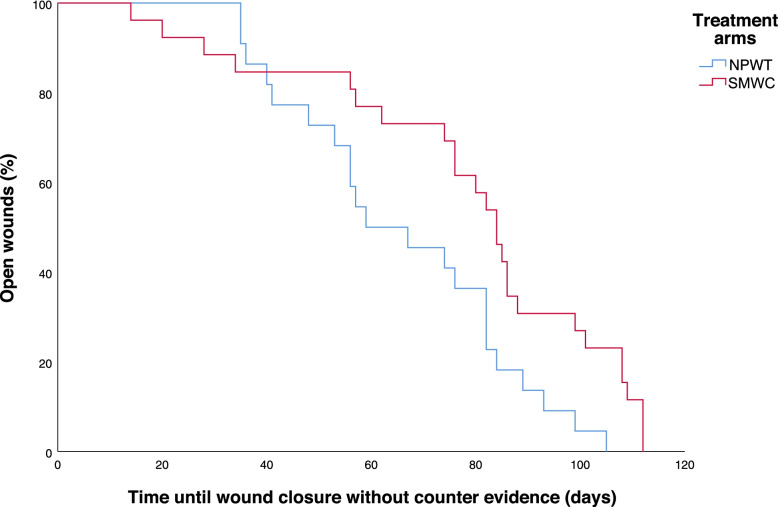
Wound closures without counter evidence within the study treatment period of 16 weeks in the PP population. Starting point of the presentation are 100% open wounds on the day of randomization / initiation of the study therapy (negative pressure wound therapy [NPWT] or standard moist wound care [SMWC]). Kaplan-Meier curves are used to show the decrease in the number of open wounds within the study treatment/observation period of 16 weeks. The course was censored for each study participant if this participant achieved wound closure without counter evidence

In the PP population, for 50% of the study participants with NPWT (22 of 44) and for 23.6% of the participants with SMWC (26 of 110) a closed wound without any counter evidence was documented within 16 weeks (difference 26.4% [95%CI 12.9 – 39.9]; Chi-squared test; *p* = 0.001) (Fig. 1). Median time to wound closure without counter evidence was significantly shorter with NPWT arm (59 days [95%CI 38.3 – 79.7] days) than with SMWC (84 days [95%CI 77.8 – 90.2]; Log-Rank-Test; *p* = 0.015). Fig. 2

### Time, human resources and material required for dressing changes

In the PP population, the mean (SD) number of dressing changes per study participant was lower with NPWT (35.1 (18.6)) than with SMWC (42.9 (21.4)) (mean difference 7.8; U test; *p* = 0.067).

NPWT dressing changes between study visits were most frequently performed in outpatient treatment facilities (full range of wound care including outpatient surgical procedures provided by physicians, nurses, nursing assistance and other personnel, e.g. podiatrists). SMWC dressing changes between study visits were most frequently performed by outpatient nursing services (home care including dressing changes and wound cleansing provided by nurses). Inpatient treatment facilities were used somewhat less frequently in both treatment arms. Detailed information on the proportion of dressing changes performed by each ambulatory care service is available in Table 3 in the Additional file 1.

The mean (SD) time required per dressing change was significantly lower with SMWC (19.7 (12.8) minutes) than with NPWT (16.5 (8.2) minutes) (U test; *p* < < 0.0001). (Additional file 1 Table 4).

Nurses (NPWT 34 of 44 (77.3%)), SMWC 92 of 109 (84.4%)), assistant physicians (NPWT 7 of 44 (15.9%), SMWC 23 of 109 (21.1%)) and specialist physicians (NPWT 21 of 44 (47.7%); SMWC 61 of 109 (56.0%)) were used more frequently in study participants of the NPWT arm. Nursing assistance was used more frequently in study participants of the SMWC arm (NPWT 3 of 44 (6.8%), SMWC (31 of 109 (28.4%)).

For 41 of 44 study participants with NPWT, the use of wound dressings and systems from KCI was documented during the study treatment phase of 16 weeks. KCI-Granufoam® black dressings and KCI-Acti V.A.C.® system were mainly used (Additional file 1 Table 5). Smith & Nephew products were not used in the PP population.

Use of an SMWC primary wound dressing (wound filler) and/or secondary wound dressing (wound cover) was documented for all study participants with SMWC and for 37 of 44 study participants with NPWT (84.1%). (Additional file 1 Table 6) For wound filler, the use of hydrogel and hydrogel-coated hydrophobic dressings was approximately equal in the two treatment arms. Alginate with and without silver, hydrofiber with and without silver, and hydrophobic materials were used more frequently in the NPWT arm. Foams with and without silver or antiseptics were used more frequently in the SMWC arm. Compresses and foams were used as wound coverings for the majority of study participants, with the aforementioned materials being used for more study participants in the SMWC arm than in the NPWT arm. A relevant proportion of study participants received hydrophobic materials for wound covering, and the use of this dressing type was more common in the NPWT arm.

### Wound cleansing and surgical debridement during the study treatment time

All study participants in the PP population underwent wound cleansing and/or decontamination at least once within 16 weeks. (Additional file 1 Table 7) The number of wound cleansings per study participant did not differ significantly between treatment arms (mean (SD) NPWT 30.2 (18.8), SMWC 29.9 (16.6); U test; *p* = 0.722). Mechanical wound cleansing (NPWT 35 of 44 (79.5%), SMWC 83 of 110 (75.5%)) and/or wound irrigation (NPWT 31 of 44 (70.5%), SMWC 70 of 110 (63.6%)) were used more frequently among study participants with NPWT. A higher proportion of study participants with SMWC received nonsurgical debridement of the wound (NPWT 17 of 44 (38.6%), SMWC 51 of 110 (46.4%)) and antiseptic use (NPWT 7 of 44 (15.9%), SMWC 29 of 110 (26.4%)).

The proportion of study participants with surgical debridement was significantly lower in the NPWT arm (3 of 44 (6.8%)) than in the SMWC arm (29 of 110 (26.4%) (Chi-squared test; *p* = 0.008). (Additional file 1 Table 8) The number of surgical debridements per study participant did not differ significantly between treatment arms (mean (SD) NPWT 6.3 (1.5), SMWC 6.1 (6.4); U test; *p* = 0.356). The mean (SD) duration per debridement was significantly shorter in the NPWT arm (5.9 (1.8)) than in the SMWC arm (33.0 (73.0)) (U test; *p* = 0.010). The duration of debridements per study participant was also shorter in the NPWT arm (20.5 (20.5)) than in the SMWC arm (43.8 (46.7)). However, the difference was not significant (*p* = 0.395, U test).

### Wound edge and wound environment protection

In the NPWT arm, 40 of 44 (90.9%) study participants and in the SMWC arm, 97 of 110 (88.2%) study participants received wound margin and wound environment protection measures. (Additional file 1 Table 9) The proportion of study participants with hyperkeratosis ablation was similar in both treatment arms (NPWT 23 of 40 (57.5%), SMWC 59 of 97 (60.8%)). Skin care procedures were used more frequently among study participants in the SMWC arm (90 of 97 (92.8%)) than in the NPWT arm (32 of 40 (80.0%)). A higher proportion of study participants in the NPWT arm (31 of 40 (77.5%)) received skin protection measures (SMWC 47 of 97 (48.5%)).

### Pressure relief

The use of pressure relief measures was documented for almost all study participants within the study treatment period of 16 weeks (NPWT 43 of 44 (97.7%), SMWC 107 of 110 (97.3%); Chi-squared test; *p* = 0.422). (Additional file 1 Table 10).

The majority of study participants received relief footwear (NPWT 30 of 42 (71.4%), SMWC 77 of 107 (72.0%)). A smaller proportion of participants used footwear with diabetes-adapted footbed (NPWT 9 of 42 (21.4%), SMWC 19 of 107 (17.8%) and orthopedic fittings (NPWT 7 of 42 (16.7%), SMWC 8 of 107 (7.5%)). Few study participants received a full-contact cast (NPWT 2 of 42 (4.8%), 4 of 107 (3.7%)). Plastic boots were not used.

Some participants used the additional support provided by walking aids (NPWT 6 of 42 (14.3%), SMWC 22 of 107 (20.6%)), wheelchairs (NPWT 6 of 42 (14.3%), SMWC 27 of 107 (25.2%)), or initial bed rest (NPWT 8 of 42 (19.0%), SMWC 20 of 107 (18.7%)).

For the majority of study participants, pressure relief could be provided completely (NPWT 23 of 43 (53.5%), SMWC 50 of 107 (46.7%)) or partially (NPWT 10 of 43 (23.3%), SMWC 30 of 107 (28.0%)) (combined complete & partial NPWT 10 of 43 (23.3%), 26 of 107 (24.3%)). For one study participant, in addition to partial pressure relief, lack of pressure relief was reported without the measure being mentioned.

### Revascularization during the study treatment time

We previously published the revascularization procedures that were performed before study start.[23].

Information on the vascular occlusion locations is available in Table 11 in the Additional file 1. During the study treatment period, 4 of 44 (9.1%) study participants with NPWT and 13 of 110 (11.8%) study participants with SMWC were revascularized (Chi-squared test; *p* = 0.626). (Additional file 1 Table 12) All documented occlusions or revascularization procedures had an impact on the direct care area of the study wound. Most study participants underwent percutaneous transluminal angioplasty (PTA) (NPWT 2 of 4 (50%), SMWC 7 of 13 (53.8%)). A smaller proportion of study participants received PTA and stenting (NPWT 1 of 4 (25%), SMWC 2 of 13 (15.4%)), venous bypass (SMWC 1 of 4 (25%), 2 of 13 (15.4%)), and thrombarterioectomy and patch plasty (NPWT 0 of 4 (0%), SMWC 1 of 13 (7.7%)). Most revascularization procedures had a sufficient outcome (NPWT *N* = 4, SMWC *N* = 12). Only in two cases per treatment arm the outcome was not assessable.

### Amputations during the study treatment time

There was no significant difference between the treatment arms in the percentage of study participants with amputation within 16 weeks (NPWT arm 10 of 44 (22.7%), SMWC arm 21 of 110 (19.1%); Chi-squared test; *p* = 0.611). (Additional file 1 Table 13) The mean (SD) duration of amputations per study participant was less in the NPWT arm (23.3 (7.5) minutes) than in the SMWC arm (36.6 (22.2) minutes) (U test; *p* = 0.126).

### Coverage of tissue defects

In the PP population, only 2 study participants from each treatment arm received defect coverage (NPWT 2 of 44 (4.5%), 2 of 110 (1.8%); Chi-squared test, *p* = 0.336). One study participant had two defect covers. For the time required for defect coverage, 2 measurements were available in the NPWT arm (total 110 minutes) and one measurement was available in the SMWC arm (30 minutes). In the NPWT arm, both study participants received a skin graft. In the SMWC arm, one study participant received a skin graft and one study participant received a local flap.

### Evaluating “real-life” with the ITT population

In the ITT population, due to treatment changes NPWT was also performed in some study participants in the SMWC arm. Care and treatment times in the ITT population were longer and differences between treatment arms were smaller. Other than in the PP population, time until hospital discharge was longer in the NPWT arm. The wound closure rate and the rate of study participants achieving 95% granulation of the wound were not significantly different between the treatment arms, but still higher with NPWT. The number of dressing changes was significantly lower with NPWT. Further details on the results of the ITT population are provided the Additional file 1 Tables 14 - 30.

## Discussion

In the DiaFu study, treatment length within 16 weeks was significantly shorter with NPWT than with SMWC, which corresponds to the previously reported finding that time to complete, verified and sustained wound closure was significantly shorter with NPWT in the PP population [23]. Furthermore, in the additional analysis on wound closure without evidence on reopening within 14 days after initial closure within 16 weeks, we demonstrated that NPWT was superior to SMWC in both wound closure rate and time to wound closure. The results of studies with diabetic foot wounds [16] and with wounds of other origins [17, 18] showing that NPWT shortens treatment time were confirmed in our study.

The study shows the typical interaction of both treatment settings (outpatient and inpatient) with the majority of study participants starting treatment in hospital and being discharged within 16 weeks. If study participants were discharged from hospital, time to first discharge was similar with NPWT and SMWC. The assumed substantial number of resources used in outpatient facilities and home care [22], was shown also to be applicable for patients with diabetic foot wounds. Length of hospital stay was relatively short in both treatment arms and one day shorter in the NPWT arm than in the SMWC arm, which corresponds to the findings of Apelqvist et al. who published an even shorter hospital stay with a similarly small difference between treatment groups [16]. Outpatient treatment time being 15 days shorter in the NPWT arm, the DiaFu study confirms previous findings that NPWT shortens outpatient resource use [16]. With approximately equal hospital (re)admission) rates, the DiaFu study, including post-amputation wounds as well as diabetic foot ulcers after debridement or wound cleansing, again confirms the results of Apelqvist et al. [16]. In contrast to other studies involving patients with only severe wounds [14], hospitalization rate seems to be less relevant for the typical mixed population of the clinical routine.

The number of dressing changes was lower in the NPWT arm, remembering that NPWT may be followed by SMWC in order to achieve complete wound closure, which by their nature are more frequent and require less effort than NPWT changes. However, the time required per dressing change was shorter in the SMWC arm. Previous studies have also shown the reduced number of dressing changes, but they have not accounted for the additional SMWC dressing changes required until complete healing or evaluated the time required for dressing changes [16, 20].

The number of wound cleansings was almost the same in the treatment arms. Nonsurgical debridement to intact tissue structures and surgical debridement were performed more frequently in the SMWC arm. The significantly lower number of surgical debridements with significantly lower time expenditure demonstrated in the DiaFu study, confirms the results of previous studies, showing NPWT to reduce resource use for surgical procedures including debridement [16]. However, little emphasis was placed on a detailed assessment of resource use for the full range of wound cleansing interventions. Resource utilization for treatment components associated with wound care, such as wound environment protection, pressure relief, and revascularization, was poorly reported in previously published studies. Although the results for the above parameters hardly differ between the treatment arms, they still need to be included in the assessment of resource utilization. Frequently, cost analyses were published without informing about the underlying resource utilization data, which complicates both the assessment of the data and its use for further analysis in the different countries of the world with different cost structures. With the resource use data provided in this article we would like to support cost analyses worldwide, which, for example, can monetarily evaluate both the costs and health outcomes of the two treatment methods in the context of a cost-benefit analysis, taking into account the actual country-specific costs of materials and personnel.

### Limitations

Our results must be interpreted in light of several limitations.

Some of these limitations, which also affect the results of the resource use assessment, have already been published when reporting the primary outcomes of the study [23]: Missing endpoint documentation, premature discontinuation of NPWT, and unapproved treatment changes negatively affected the wound closure treatment outcome and may have caused bias in the results. Due to protocol violations, a high number of study participants had to be excluded from the PP population, which resulted in largely different number of participants in the treatment arms. Using the criteria for complete, verified and for a minimum of 14 days sustained wound closures as set in the study protocol, more than 50% of the wounds were still open after 16 weeks. To address this, an additional post hoc analysis of wound closures without counter evidence within 132 days was performed. Even though this approach, which excluded the influence of documentation errors, allowed the calculation of medians, a large number of wounds were not closed after 16 weeks. The evaluation is limited to 16 weeks. Treatment times of the studies that formed the basis for establishing 16 weeks of treatment time were not sufficient for study participants in the DiaFu study, which observed routine clinical practice [19, 24].

Further limitations relate specifically to resource utilization: First, the present study did not collect data on how many of the wounds were closed by means of secondary healing or with surgical measures. Second, no separate evaluation of personnel and material use in the hospital and during outpatient care, but only an evaluation of the use of in- and outpatient treatment facilities and nursing services for dressing changes was performed. Furthermore, the exemplary recording of the time spent on dressing changes in the study sites, which were outpatient and inpatient treatment facilities, may have led to a bias in the survey of personnel use, as the personnel composition in nursing services is different from that in treatment facilities. Third, intensive care unit days were not evaluated in the DiaFu-study. Fourth, no formal cost analysis was performed. For an adequate cost analysis, the entire course of the patient should be considered starting with the first appearance of the ulcer until healing or within a defined period of time. In the present study, the inclusion criterion was an ulcer time of at least 4 weeks. The exact length of existence could often not be determined, and interventions that did not occur within the period between screening and randomization were not included in the analysis. Furthermore, the economic status of different countries is a major problem when analyzing disease state cost [22]. Unlike previous studies, which typically present country-specific unit costs for personnel, inpatient accommodations, and supplies based on the prevailing billing system, our study presents resource use results that can be used as the basis for any economic analysis worldwide. Information on optimal material use for subsequent cost analyses needs to be derived from other resources.

## Conclusions

The aim of this evaluation, to compare resource utilization of negative pressure wound therapy (NPWT) and standard moist wound care (SMWC) for diabetic foot wounds after amputation, surgical debridement or wound cleansing was achieved. The results of the DiaFu study in terms of shorter treatment duration, shorter ambulatory care time, less time spent on surgical debridements, and fewer dressing changes suggest that NPWT is an efficient treatment alternative to SMWC and confirm the experience from previous studies. However, the time required per dressing change was lower with SMWC. However, previously published clinical results from the DiaFu trial showed that NPWT was not superior to SMWC for diabetic foot wounds and that the overall closure rate was low due to documentation deficiencies and deviations from treatment guidelines that negatively affected wound closure outcomes. The additional analysis performed on wound closure without evidence of reopening within 14 days, which showed that NPWT achieves more frequent and faster wound closure, can only partially remedy this situation, since the a priori defined quality criteria for documentation were no longer met.

The DiaFu study is the first RCT to examine resource utilization for diabetic foot care with NPWT in such detail, providing valuable data on personnel and material use and procedures associated with wound treatment for subsequent cost analyses.

## Supplementary Information

**Additional file 1.**

## Data Availability

The datasets used and/or analyzed during the current study are available from the corresponding author on reasonable request.

## Abbreviations

NPWT: Negative pressure wound therapy
SMWC: Standard moist wound care
PP: Per protocol
ITT: Intention-to-treat (ITT)
W.H.A.T: Wound Healing Analyzing Tool
KCI: Kinetic Concepts Incorporated
S&N: Smith & Nephew
SD: Standard deviation
CI: Confidence interval
Min: Minimum
Max: Maximum
NA: Not applicable
CONSORT: Consolidated Standards of Reporting Trials
PTA: Percutaneous transluminal angioplasty
