# Supplementary material for: NPWT resource use compared with standard moist wound care in diabetic foot wounds: DiaFu randomized clinical trial results
Source: J Foot Ankle Res. 2022 Sep 30;15:72. doi: 10.1186/s13047-022-00569-w (PMC9524075; doi:10.1186/s13047-022-00569-w)
Supplement: Supplementary file 1 — Additional file 1. [file 13047_2022_569_MOESM1_ESM.docx]

Additional file 1

PP population

# Table 1: Hospital discharge of study participants with inpatient study start, outpatient treatment continuation and wound closures until the end of the maximum study treatment time of 16 weeks in the PP population

| **Randomized treatments arms / statistical test** | **NPWT** | **SMWC** | **P-value**  **(Test)** |
| --- | --- | --- | --- |
| Study participants in the PP population, No. | 44 | 110 | NA |
| Study participants with outpatient study start, No. (%) | 10 (22.7) | 29 (26.4) | 0.639  (Chi²) |
| Study participants with study start in hospital, No. (%) | 34 (77.3) | 81 (73.6) |  |
| Study participants with study start in hospital and discharged within 16 weeks, No. (%) | 33 (97.1) | 80 (98.8) | 0.523  (Chi²) |
| Time until first discharge from hospital during the study treatment period of 42 days (days) |  |  |  |
| Mean (SD) | 10.1 (8.8) | 11.5 (13.8) | 0.825  (U) |
| Min-Max | 2 – 37 | 1 – 75 |  |
| Study participants with treatment continuation after hospital discharge, No. (%)  *For one study participants with NPWT no data were available after hospital discharge. | 32 of 33* (97.0) | 80 of 80 (100) | NA |
| Study participants with **NPWT after hospital discharge**, No. (%) | 15 of 32 (46.9) | 0 (0) | NA |
| Length of NPWT after hospital discharge (days) |  |  |  |
| Mean (SD) | 42.3 (31.0) | NA | NA |
| Min - Max | 5 – 107 |  |  |
| Study participants with **NPWT and SMWC after hospital discharge** | 13 (40.6) | 0 | NA |
| Length of SMWC additionally to NPWT after hospital discharge (days) |  |  |  |
| Mean (SD) | 36.5 (23.0) | NA | NA |
| Min - Max | 0 – 72 |  |  |
| Study participants with NPWT after hospital discharge and hospital readmission, No. (%) | 5 (33.3) | NA | NA |
| Study participants with NPWT after hospital discharge and wound closure without counter evidence, No. (%) [95% CI] | 8 (53.3)  [28.1-78.6] | 0 | NA |
| Time until wound closure without counter evidence for study participants with NPWT after hospital discharge |  |  |  |
| Mean (SE) | 78.0 (9.6) | NA | NA |
| [95% KI] | [62.8-93.2] |  |  |
| Study participants with **SMWC only after hospital discharge**, No. (%) | 17 (53.1) | 80 (100) | NA |
| Length of SMWC after hospital discharge (days) |  |  |  |
| Mean (SD) | 79.9 (29.2) | 87.5 (25.2) | NA |
| Min - Max | 18 – 109 | 10 – 110 |  |
| Study participants with SMWC only after hospital discharge and hospital readmission, No. (%) | 5 (29.4) | 20 (25.0) | NA |
| Study participants with SMWC only after hospital discharge and wound closure without counter evidence, No. (%) [95% CI] | 8 (53.3)  [28.1-78.6] | 17 (21.3)  [12.3-30.2] | NA |
| Time until wound closure for study participants with SMWC only after hospital discharge |  |  |  |
| Mean (SE) | 68.1 (24.3) | 81.8 (24.8) | NA |
| [95% CI] | [47.8-88.5] | [69.1-94.6] |  |
| Study participants with inpatient study start, hospital discharge and readmission, No. (%) | 10 of 34 (29.4) | 20 of 81 (24.7) | NA |
| Study participants with outpatient study start and hospital admission, No. (%) | 1 of 10 (10.0) | 7 of 29 (24.1) | NA |

# Table 2: Local wound treatment within the follow-up period in the PP population

| **Randomized treatments arms / statistical test** | **NPWT** | **SMWC** |
| --- | --- | --- |
| Study participants in the PP population, No. | 44 | 110 |
| Study participants with available data on local wound treatment during the follow-up period, No. | 37 | 96 |
| Study participants without local wound treatment during the follow-up period, No. (%) | 21 of 37 (56.8) | 38 of 96 (39.6) |
| Study participants with local wound treatment during the follow-up period, No. (%) | 16 of 37 (43.2) | 58 of 96 (60.4) |
| Study participants with data on type of local wound treatment, No. | 16 | 58 |
| Study participants with NPWT during the follow-up period, No. (%) | 1 of 16 (6.3) | 2 of 58 (3.4) |
| Study participants with SMWC during the follow-up period, No. (%) | 15 of 16 (93.8) | 56 of 58 (96.6) |
| Study participants with data on start and end of local wound treatment during the follow-up period, No. | 5 | 17 |
| Length of local wound treatment during the follow-up period (days) |  |  |
| Mean (SD) | 118.6 (71.4) | 119.7 (70.6) |
| Min - Max | 15 - 199 | 1 - 189 |
| Study participants with NPWT and data on start and end of local wound treatment during the follow-up period, No. | 0 | 1 |
| Length of NPWT during the follow-up period (days) |  |  |
| Mean (SD) | NA | 20 (NA) |
| Min - Max | NA | 20 - 20 |
| Study participants with SMWC and data on start and end of local wound therapy during the follow-up period, No. | 5 | 16 |
| Length of NPWT during the follow-up period (days) |  |  |
| Mean (SD) | 118.6 (71.4) | 125.9 (68.0) |
| Min - Max | 15 - 199 | 1 - 189 |

# Table 3: Dressing changes within 16 weeks in the PP population

| **Randomized treatments arms / statistical test** | **NPWT** | **SMWC** | **P-value**  **(Test)** |
| --- | --- | --- | --- |
| Study participants in the PP population, No. | 44 | 110 | NA |
| Study participants without dressing changes, No. | 1 | 1 | NA |
| Study participants with dressing changes, No. | 43 | 109 | NA |
| Study participants with at least one missing record on the number of dressing changes between study visits, No. | 3 | 7 | NA |
| Study participants with at least one record on the number of dressing changes between study visits, No. | 43 | 109 | NA |
| Number of dressing changes per study participant |  |  |  |
| Mean (SD) | 35.1 (18.6) | 42.9 (21.4) | 0.067  (U) |
| Min – Max | 2 - 75 | 2 - 115 |  |
| Study participants with at least one missing personnel record for dressing changes between study visits, No. | 1 | 0 | NA |
| Study participants with at least one personnel record for dressing changes between study visits, No. | 43 | 109 | NA |
| Study participants with **outpatient nursing service** involvement in dressing changes between study visits, No. | 21 | 59 | NA |
| Dressing changes with outpatient nursing service involvement per study participant |  |  |  |
| Mean (SD) | 15.9 (12.4) | 24.8 (16.0) | NA |
| Min – Max | 2 - 42 | 2 - 65 |  |
| Study participants with **outpatient treatment facility** involvement in dressing changes between study visits, No. | 28 | 62 | NA |
| Dressing changes with outpatient treatment facility involvement per study participant |  |  |  |
| Mean (SD) | 16.7 (12.2) | 18.3 (12.2) | NA |
| Min – Max | 1 - 44 | 2 -50 |  |
| Study participants with **inpatient treatment facility** involvement in dressing changes between study visits, No. | 33 | 77 | NA |
| Dressing changes with inpatient treatment facility involvement per study participant |  |  |  |
| Mean (SD) | 10.5 (9.3) | 11.5 (13.9) | NA |
| Min – Max | 1 - 31 | 1 - 82 |  |
| Study participants with other personnel involved in dressing changes between study visits, N | 10 | 23 | NA |
| Dressing changes with other personnel involved per study participant |  |  |  |
| Mean (SD) | 11.6 (12.7) | 22.5 (17.0) | NA |
| Min – Max | 2 - 44 | 3 - 61 |  |

# Table 4: Exemplary record of time and personnel spent on dressing changes at the current study visit within the study treatment period of 16 weeks in the PP population

| **Randomized treatments arms / statistical test** | **NPWT** | **SMWC** | **P-value**  **(Test)** |
| --- | --- | --- | --- |
| Study participants in the PP population, No. | 44 | 110 | NA |
| Study participants with at least one documented dressing change during a study visit, No. | 44 | 110 | NA |
| **Study participants with at least one time record for dressing changes during a study visit, No.** | **44** | **110** | **NA** |
| Study participants with at least one missing time record for dressing changes during a study visit, No. | 1 | 4 | NA |
| Time required per dressing change |  |  |  |
| Mean (SD) | 19.7 (12.8) | 16.5 (8.2) | <0.0001  (U) |
| Min - Max | 1 - 100 | 3 - 60 |  |
| **Study participants with at least one personnel record** **for dressing changes during a study visit, No.** | **44** | **109** | NA |
| Study participants with at least one missing personnel record for dressing changes during a study visit, No. | 2 | 3 | NA |
| Study participants with **nursing assistance** contacts for dressing changes within the study visit, No. (%) | 3 of 44 (6.8) | 31 of 109 (28.4) | NA |
| Study participants with **nurse** contacts for dressing changes within the study visit, No. (%) | 34 of 44 (77.3) | 92 of 109 (84.4) | NA |
| Study participants with **assistant physician** contacts for dressing changes within the study visit, No. (%) | 7 of 44 (15.9) | 23 of 109 (21.1) | NA |
| Study participants with **specialist physician** contacts for dressing changes within the study visit, No. (%) | 21 of 44 (47.7) | 61 of 109 (56.0) | NA |
| Study participants with **other personnel** contacts for dressing changes within the study visit, No. (%) | 9 of 44 (20.5) | 19 of 109 (17.4) | NA |

# Table 5: Use of NPWT wound dressings and systems within the study treatment period of 16 weeks in the PP population

| **Randomized treatments arms** | **NPWT** | **SMWC** |
| --- | --- | --- |
| Study participants in the PP population, No. | 44 | 110 |
| **Study participants with data on the use of NPWT wound dressings, No.** | **41** | **0** |
| Study participants with KCI-Granufoam^®^black, No. | 34 | 0 |
| Study participants with KCI-White Foam^®^, No. | 5 | 0 |
| Study participants with KCI-Granufoam^®^black and KCI-White Foam^®^, No. | 1 | 0 |
| Study participants with KCI-Silver^®^ and KCI-White Foam^®^, No. | 1 | 0 |
| **Study participants with data on the use of NPWT systems, No.** | **41** | **0** |
| Study participants with KCI-V.A.C. Freedom^®^, No. | 3 | 0 |
| Study participants with KCI-Acti V.A.C.^®^, No. | 33 | 0 |
| Study participants with KCI-V.A.C. Freedom^®^ and KCI-Acti V.A.C.^®^, No. | 1 | 0 |
| Study participants with KCI-V.A.C. Freedom^®^ and KCI-INFO V.A.C.^®^, No. | 1 | 0 |
| Study participants with KCI-Acti V.A.C.^®^ and KCI-INFO V.A.C.^®^, No. | 3 | 0 |

# Table 6: Use of SMWC dressings within the study treatment period of 16 weeks in the PP population

| **Randomized treatments arms** | **NPWT** | **SMWC** |
| --- | --- | --- |
| Study participants in the PP population, No. | 44 | 110 |
| Study participants at least once treated with SMWC, No. (%) | 38 (86.4) | 110 (100) |
| Study participants with at least one documentation of a wound dressing (wound filler or wound cover*) within the study treatment period, No. (%) | 37 (84.1) | 110 (100) |
| **Study participants with at least one documentation of a wound filler, N** | 32 | 109 |
| Hydrogel and hydrogel coated hydrophobic dressings, No. (%) | 16 of 32 (50%) | 56 of 109 (51.4%) |
| Hydrogel, No. | 15 | 48 |
| Hydrogel coated hydrophobic dressings, No. | 1 | 8 |
| Alginate with and without silver, No. (%) | 14 of 32 (43.8%) | 42 of 109 (38.5%) |
| Alginate, No. | 9 | 31 |
| Alginate with silver, No. | 5 | 11 |
| Hydrofiber with and without silver, No. (%) | 10 of 32 (31.3%) | 24 of 109 (22.0%) |
| Hydrofiber, No. | 7 | 19 |
| Hydrofiber with silver, No. | 3 | 5 |
| Foams with and without silver or antiseptics, No. (%) | 8 of 32 (25.0%) | 39 of 109 (35.8%) |
| Polyurethane (PU) foam, tamponade, cavity, No. | 8 | 39 |
| Polyurethane (PU) foam, tamponade, cavity with silver, No. | 0 | 0 |
| Polyurethane (PU) foam, tamponade, cavity with antiseptics, No. | 0 | 0 |
| Collagen, No. (%) | 4 of 32 (12.5%) | 2 of 109 (1.8%) |
| Hyaluronic acid, No. (%) | 0 of 32 (0%) | 0 of 109 (0%) |
| Hydrophobic materials, No. (%) | 13 of 32 (40.6%) | 34 of 109 (31.2%) |
| Silver dressing materials with activated carbon, No. (%) | 0 of 32 (0%) | 5 of 109 (4.6%) |
| Silver dressing materials without activated carbon, No. (%) | 2 of 32 (6.3%) | 3 of 109 (2.8%) |
| Gauze and fleece compresses, tamponades partially soaked with antiseptics, No. (%) | 0 of 32 (0%) | 7 of 109 (6.4%) |
| Gauze partially soaked with antiseptic or hemostatic agent, No. (%) | 0 of 32 (0%) | 0 of 109 (0%) |
| Other indications which, by definition, are not wound fillers or cannot be clearly assigned to a category, No. (%) | 0 of 32 (0%) | 3 of 109 (2.8%) |
| Maggots / Maggot therapy, No. | 0 | 1 |
| Suprasorb, No. | 0 | 2 |
| Other wound filler without specification, No. (%) | 2 of 32 (6.3%) | 6 of 109 (5.5%) |
| **Study participants with at least one documentation of a wound cover** | 37 | 110 |
| Gauze compresses, No. (%) | 23 of 37 (62.2%) | 75 of 110 (68.2%) |
| Nonwoven compresses, No. (%) | 3 of 37 (8.1%) | 14 of 110 (12.7%) |
| Absorbent compresses with cellulose core partially activated with Ringer's solution, No. (%) | 8 of 37 (21.6%) | 39 of 110 (35.5%) |
| Absorbent compresses with cellulose core, No. | 7 | 36 |
| Absorbent compresses with cellulose core activated with Ringer's solution, No. | 1 | 3 |
| Superabsorbent dressings, No. (%) | 3 of 37 (8.1%) | 8 of 110 (7.3%) |
| Films sterile and non-sterile, No. (%) | 0 of 37 (0%) | 2 of 110 (1.8%) |
| Films sterile, No. | 0 | 0 |
| Films non-sterile, No. | 0 | 2 |
| Hydrocolloids, No. (%) | 3 of 37 (8.1%) | 2 of 110 (1.8%) |
| Hydrofiber with and without silver, No. (%) | 0 of 37 (0%) | 2 of 110 (1.8%) |
| Adhesive hydrofiber, No. | 0 | 0 |
| Non-adhesive hydrofiber, No. | 0 | 1 |
| Non-adhesive hydrofiber with silver, No. | 0 | 1 |
| Foams with and without ibuprofen or silver, No. (%) | 17 of 37 (45.9%) | 75 of 110 (68.2%) |
| PU foam adhesive, No. | 4 | 18 |
| PU foam soft adhesive, No. | 6 | 27 |
| PU foam non-adhesive, No. | 7 | 29 |
| PU foam non-adhesive with Ibuprofen, No. | 0 | 1 |
| PU foam non-adhesive with silver, No. | 0 | 0 |
| Odor-reducing dressing materials with activated carbon, No. (%) | 0 of 37 (0%) | 0 of 110 (0%) |
| Hydrophobic materials with and without silver or honey | 8 of 37 (21.6%) | 21 of 110 (19.1%) |
| Hydrophobic materials, No. | 8 | 19 |
| Hydrophobic materials with silver, No. | 0 | 1 |
| Hydrophobic materials with honey, No. | 0 | 1 |
| Silver dressings with activated charcoal, No. (%) | 1 of 37 (2.7%) | 5 of 110 (4.5%) |
| Silver dressings without activated charcoal, No. (%) | 1 of 37 (2.7%) | 1 of 110 (0.9%) |
| Other material without direct effect on the wound / additives, No. (%) | 8 of 37 (21.6%) | 37 of 110 (33.6%) |
| Cushioning material and bandages, fixatives, compression bandages and abdominal sheets, No. | 2 | 18 |
| Steri-strips, No. | 1 | 2 |
| Plaster, No. | 1 | 10 |
| Additive of antiseptics without specification of the dressing, No. | 3 | 6 |
| Protection, No.: | 1 | 1 |
| *Mepilex Border for skin protection, No.* | *0* | *1* |
| *for protection, No.* | *1* | *0* |
| Other wound covers without specification, No. (%) | 1 of 37 (2.7%) | 7 of 110 (6.4%) |

*Multiple answers were possible

# Table 7: Wound cleansing and decontamination within the study treatment period of 16 weeks in the PP population

| **Randomized treatments arms / statistical test** | **NPWT** | **SMWC** | **p value**  **(test)** |
| --- | --- | --- | --- |
| Study participants in the PP population, No. | 44 | 110 | NA |
| Study participants without wound cleansing or decontamination, No. (%) | 0 of 44 (0%) | 0 of 110 (0%) | NA |
| Study participants with wound cleansing or decontamination, No. (%) | 44 (100%) | 110 (100%) | NA |
| Number of wound cleansings and decontaminations | 1314 | 3330 | NA |
| Wound cleansings and decontaminations per study participant  Mean (SD)  Min - Max | 29.9 (16.6)  1 - 64 | 30.3 (19.7)  5 – 91 | 0.722  (U) |
| **Type of wound cleansing and decontamination*** |  |  |  |
| Study participants with debridement to intact anatomic structures, No. (%) | 17 (38.6) | 51 (46.4) | NA |
| Study participants with mechanical wound cleansing, No. (%) | 35 (79.5) | 83 (75.5) | NA |
| Study participants with wound irrigation, No. (%) | 31 (70.5) | 70 (63.6) | NA |
| Study participants with wrappings and long-term wet periods, No. (%) | 2 (4.5) | 4 (3.6) | NA |
| Study participants with use of antiseptics, No. (%) | 7 (15.9) | 29 (26.4) | NA |
| Study participants with ultrasonic cleaning, No. (%) | 0 (0) | 0 (0%) | NA |
| Study participants using moisturizing materials, No. (%) | 0 (0) | 2 (1.8) | NA |
| Study participants with enzymatic wound cleansing, No. (%) | 0 (0) | 1 (0.9) | NA |
| Study participants with wound cleaning by fly maggots, No. (%) | 2 (4.5) | 4 (3.6) | NA |
| Study participants with osmotic wound cleansing, No. (%) | 0 (0) | 0 (0) | NA |
| Study participants with other wound cleansing and decontamination methods, No. (%) | 2 (4.5) | 5 (4.5) | NA |

*Multiple answers were possible

# Table 8: Surgical debridement within the study treatment period of 16 weeks in the PP population

| **Randomized treatments arms /statistical test** | **NPWT** | **SMWC** | **p value**  **(test)** |
| --- | --- | --- | --- |
| Study participants in the PP population, No. | 44 | 110 | NA |
| Study participants without debridement, No. (%) | 41 of 44 (93.2) | 81 of 44 (73.6) | 0.008  (Chi^2^) |
| Study participants with debridement, No. (%) | 3 of 44 (6.8%) | 29 of 110 (26.4%) |  |
| Number of debridements | 19 | 177 | NA |
| Debridements per study participant |  |  |  |
| Mean (SD) | 6.3 (1.5) | 6.1 (6.4) | 0.356  (U) |
| Min - Max | 5 - 8 | 1 - 25 |  |
| Study participants with available total duration of debridements, No. | 2 | 26 | NA |
| Number of debridements with time expenditure | 9 | 89 | NA |
| *Study participants with at least one missing indication of debridement duration, No.* | *2* | *6* | NA |
| *Missing information on the duration of debridement, No.* | *6* | *18* | NA |
| Time spent per debridement |  |  |  |
| Mean (SD) | 5.9 (1.8) | 33.0 (73.0) | **0.010**  **(U)** |
| Min - Max | 5 - 9 | 2 - 490 |  |
| Time spent for debridements per study participant |  |  |  |
| Mean (SD) | 20.5 (20.5) | 43.8 (46.7) | 0.395  (U) |
| Min - Max | 6 - 35 | 5 - 230 |  |

# Table 9: Wound margin and wound environment protection implemented within the study treatment period of 16 weeks in the PP population

| **Randomized treatments arms / statistical test** | **NPWT** | **SMWC** | **p value**  **(test)** |
| --- | --- | --- | --- |
| Study participants in the PP population, No. | 44 | 110 | NA |
| Study participants with wound margin and environment protection measures, No. (%) | 40 of 44 (90.9%) | 97 of 110 (88.2%) | 0.626  (Chi^2^) |
| **Type of protective measures*:** |  |  |  |
| Study participants with hyperkeratosis ablation, No. (%) | 23 of 40 (57.5%) | 59 of 97 (60.8%) | NA |
| Study participants with skin care, No. (%) | 32 of 40 (80.0%) | 90 of 97 (92.8%) | NA |
| Study participants with skin protection, No. (%) | 31 of 40 (77.5%) | 47 of 97 (48.5%) | NA |
| Study participants with mycoses therapy, No. (%) | 0 of 40 (0%) | 0 of 97 (0%) | NA |
| Study participants with measures against contact allergy, No. (%) | 0 of 40 (0%) | 0 of 97 (0%) | NA |
| Study participants with other measures, No. (%) | 4 of 40 (10.0%) | 7 of 97 (7.2%) | NA |

*Multiple answers were possible

# Table 10: Pressure relief within the study period of 16 weeks in the PP population

| **Randomized treatments arms** | **NPWT** | **SMWC** |
| --- | --- | --- |
| Study participants in the PP population, No. | 44 | 110 |
| Study participants without data on pressure relief, No. (%) | 1 (2.3%) | 3 (2.7%) |
| Study participants with pressure relief measures implemented, No. (%) | 43 (97.7%) | 107 (97.3%) |
| Study participants with pressure relief measures implemented, but at least once without indication of type of protective measure, No. | 3 | 5 |
| Study participants with pressure relief measures implemented, but at least once without indication of type of protective measure but with indication if pressure relief was ensured, No. | 3 | 5 |
| Study participants with pressure relief measures implemented and at least one indication of the type of protective measure, No. (%) | 42 of 43 (97.7%) | 107 of 107 (100%) |
| **Study participants with the respective type of pressure relief measure:** |  |  |
| Protective footwear: relief shoes, No. (%) | 30 of 42 (71.4%) | 77 of 107 (72.0%) |
| Protective footwear: interim shoes, No. (%) | 5 of 42 (11.9%) | 15 of 107 (14.0%) |
| Footwear with diabetes-adapted footbed, No. (%) | 9 of 42 (21.4%) | 19 of 107 (17.8%) |
| Orthopedic fittings, No. (%) | 7 of 42 (16.7%) | 8 of 107 (7.5%) |
| Total-Contact cast, No. (%) | 2 of 42 (4.8%) | 4 of 107 (3.7%) |
| Scotch cast boots, No. (%) | 0 of 42 (0%) | 0 of 107 (0%) |
| Ready-made or custom-made orthoses, No. (%) | 7 of 42 (16.7%) | 21 of 107 (19.6%) |
| Walking aids, No. (%) | 6 of 42 (14.3%) | 22 of 107 (20.6%) |
| Wheel chair, No. (%) | 6 of 42 (14.3%) | 27 of 107 (25.2%) |
| Initial bed rest, No. (%) | 8 of 42 (19.0%) | 20 of 107 (18.7%) |
| Other: Positioning or positioning material, No. (%) | 0 of 42 (0%) | 0 of 107 (0%) |
| Other: description of non-established methods: cut out Crocs, No. (%) | 0 of 42 (0%) | 1 of 107 (0.9%) |
| **Ensuring pressure relief per study participant:** |  |  |
| Complete, No. (%) | 23 of 43 (53.5%) | 50 of 107 (46.7%) |
| Partial, No. (%) | 10 of 43 (23.3%) | 30 of 107 (28.0%) |
| Not at all, No. (%) | 0 of 43 (0%) | 0 of 107 (0%) |
| Complete & partial, No. (%) | 10 of 43 (23.3%) | 26 of 107 (24.3%) |
| Partial & not at all, No. (%) | 0 of 43 (0%) | 1^#^ of 107 (0.9%) |

*Multiple answers were possible; #without indication the type of the pressure relief measure

# Table 11: Vascular occlusion locations within the study treatment period of 16 weeks in the PP population

| **Randomized treatments arms** | **NPWT** | **SMWC** |
| --- | --- | --- |
| Study participants in the PP population, No. | 44 | 110 |
| Study participants with central occlusion, No. | 0 | 0 |
| Study participants with inguinal occlusion, No. | 0 | 0 |
| Study participants with occlusion combination: femoral, popliteal, crural, No. | 1 | 0 |
| Study participants with occlusion combination: femoral, popliteal, pedal, No. | 0 | 1 |
| Study participants with occlusion combination: femoral, popliteal, No. | 1 | 3 |
| Study participants with occlusion combination: femoral, crural, No. | 1 | 5 |
| Study participants with occlusion combination: femoral, pedal, No. | 0 | 1 |
| Study participants with femoral occlusion, No. | 0 | 1 |
| Study participants with occlusion combination: popliteal, crural, No. | 0 | 1 |
| Study participants with popliteal occlusion, No. | 0 | 0 |
| Study participants with occlusion combination: crural, pedal, No. | 0 | 0 |
| Study participants with crural occlusion, No. | 1 | 3 |

# Table 12: Revascularization within the study treatment period of 16 weeks in the PP population

| **Randomized treatments arms** | **NPWT** | **SMWC** | **p value**  **(test)** |
| --- | --- | --- | --- |
| Study participants in the PP population, No. | 44 | 110 | NA |
| Study participants with at least one revascularization, No. (%) | 4 (9.1) | 13 (11.8) | 0.626  (Chi^2^) |
| Study participants with one revascularization, No. | 2 | 11 | NA |
| Study participants with two revascularizations, No. | 1 | 2 | NA |
| Study participants with three revascularizations, No. | 0 | 0 | NA |
| Study participants with four revascularizations, No. | 1 | 0 | NA |
| Number of revascularizations | 8 | 15 | NA |
| Revascularizations per study participant |  |  |  |
| Mean (SD)  Min - Max | 0.2 (0.7)  0 - 4 | 0.1 (0.4)  0 - 2 | 0.680  (U) |
| Study participants with at least one revascularization procedure performed with effect on the study wound, No. | 4 | 13 | NA |
| Study participants with at least one revascularization procedure performed without effect on the study wound, No. | 0 | 0 | NA |
| **Type of revascularization:** |  |  |  |
| Study participants with multilevel reconstruction, No. | 0 | 0 | NA |
| Study participants with thrombarterioectomy (TEA) and patchplasty, No. | 0 | 1 | NA |
| Study participants with polytetrafluoroethylene (PTFE) bypass graft, No. | 0 | 0 | NA |
| Study participants with venous bypass, No. | 0 | 2 | NA |
| Study participants with percutaneous transluminal angioplasty (PTA), No. | 1 | 5 | NA |
| Study participants with the sequence: PTA & stent, venous bypass, PTA & stent, PTA & stent, No. | 1 | 0 | NA |
| Study participants with the sequence: PTA & Stent, PTA, No. | 0 | 0 | NA |
| Study participants with the sequence: PTA & Stent, No. | 0 | 2 | NA |
| Study participants with the sequence: PTA, venous bypass, No. | 0 | 0 | NA |
| Study participants with the sequence: PTA, PTA, No. | 1 | 2 | NA |
| Study participants with other techniques, No. | 1 | 1 | NA |
| **Revascularization result:** |  |  | NA |
| Study participants with sufficient revascularization result, No. | 3 | 12 | NA |
| Study participants with insufficient revascularization result, No. | 0 | 0 | NA |
| Study participants with sufficient revascularization result and non-assessable result, No. | 1 | 1 | NA |

# Table 13: Amputations within the study treatment period of 16 weeks in the PP population

| **Randomized treatments arms** | **NPWT** | **SMWC** | **p value**  **(test)** |
| --- | --- | --- | --- |
| Study participants in the PP population, No. | 44 | 110 | NA |
| Study participants without amputation, No. (%) | 35 (79.5) | 89 (80.9) | 0.611  (Chi^2^) |
| Study participants with amputation, No. | 10 (22.7%) | 21 (19.1%) |  |
| Study participants with data on number of amputations, No. | 10 | 21 | NA |
| Number of amputations | 11 | 31 | NA |
| Amputations per study participant |  |  |  |
| Mean (SD) | 1.1 (0.3) | 1.5 (0.7) | 0.201  (U) |
| Min - Max | 1.0 – 2.0 | 1.0 – 3.0 |  |
| Study participants with data on time spent for amputations, No. | 9 | 19 | NA |
| Study participants with at least one missing data on time spent for amputations, No. | 1 | 3 | NA |
| Number of missing data for time spent for amputations | 1 | 3 | NA |
| Time spent for amputations per study participant |  |  |  |
| Mean (SD) | 23.3 (7.5) | 36.6 (22.2) | 0.126  (U) |
| Min - Max | 15 - 40 | 15 - 90 |  |

ITT population

# Table 14. Demographics, wound size, revascularization and wound surgery before study start in the ITT population

| **Baseline parameter of the PP population** | **NPWT** | **SMWC** |
| --- | --- | --- |
| Study participants in the ITT population, No. | 171 | 174 |
| **Age in years,** Mean (SD) | 67.6 (12.3) | 68.1 (11.5) |
| **Sex,** No. (%) |  |  |
| Male | 133 (77.8) | 134 (77.0) |
| Female | 38 (22.2) | 40 (23.0) |
| **Wound surface area at randomization** **calculated from CRF entries (width and length),** mm^2^ |  |  |
| Study participants with data available (used from screening), No. | 171 (2) | 174 (0) |
| Mean (SD) | 1060 (1536) | 1141 (3247) |
| Min-Max | 20 – 13 188 | 12 – 40 773 |
| **Wound volume at randomization calculated from CRF entries (width, length and depth),** mm^3^ |  |  |
| Study participants with data available (used from screening), No. |  |  |
| Mean (SD) | 22498 (58930) | 21740 (74181) |
| Min-Max | 0 – 60 2880 | 0 – 81 5458 |
| **Study participants with revascularization before study start, No. (%)** | 9 (5.3) | 14 (8.0) |
| Study participants with Percutaneous Transluminal Angioplasty (PTA), No. (%) | 6 of 9 (67) | 7 of 14 (50) |
| Study participants with PTA and Stent, No. (%) | 0 of 9 (0) | 1 of 14 (7) |
| Study participants with Venous Bypass, No. (%) | 2 of 9 (22) | 3 of 14 (21) |
| Study participants with Polytetrafluoroethylene Bypass, No. (%) | 0 of 9 (0) | 1 of 14 (7) |
| Study participants with Thromboendarterectomy, No. (%) | 0 of 9 (0) | 0 of 14 (0) |
| Study participants with Thromboendarterectomy and Patch plastic, No. (%) | 0 of 9 (0) | 14% (2 of 14) |
| Study participants with multi-level reconstruction, No. (%) | 0 of 9 (0) | 0 of 14 (0) |
| **Study participants with wound surgery before treatment start, No. (%)** |  |  |
| Study participants with surgical debridement, No. (%) | 74 (43.3) | 72 (41.4) |
| Study participants with major amputations, No. (%) | 0 (0) | 0 (0) |
| Study participants with minor amputations, No. (%) | 16 (9.4) | 33 (19.0) |
| Study participants with minor amputations in the border zone to vital tissues, necrosectomy and debridement, No. (%) | 37 (21.6) | 30 (17.2) |

# Table 15. Care status and treatment periods during the active study treatment time of 16 weeks in the ITT population

| **Randomized treatments arms / statistical test** | **NPWT** | **SMWC** | **p value (Test)** |
| --- | --- | --- | --- |
| Study participants in the ITT population, No. | 171 | 174 | NA |
| Study participants with inpatient study start, No. (%) | 127 (74.3%) | 130 (74.7%) | 0.925  (Chi^2^) |
| Study participants with outpatient study start, No. (%) | 44 (25.7%) | 44 (25.3%) |  |
| **Length of care (days)** |  |  |  |
| N | 171 | 174 |  |
| Mean (SD) | 80.7 (35.6) | 88.2 (34.6) | 0.031  (U) |
| Min-Max | 5 – 112 | 3 – 112 |  |
| **Study participants with inpatient care, No. (%)** | 139 (81.3) | 144 (82.8) | 0.722  (Chi^2^) |
| **Study participants exclusively treated inpatient, No. (%)** | 8 (4.7) | 10 (5.7) | 0.655  (Chi^2^) |
| **Length of hospital stay (days)** |  |  |  |
| Mean (SD) | 21.9 (17.3) | 22.2 (23.5) | 0.122  (U) |
| Min-Max | 1 – 99 | 1 – 112 |  |
| **Study participants with outpatient care, No. (%)** | 163 (95.3) | 164 (94.3) | 0.655  (Chi^2^) |
| **Study participants exclusively treated outpatient, No. (%)** | 32 (18.7) | 30 (17.2) | 0.722  (Chi^2^) |
| **Length of outpatient care (days)** |  |  |  |
| Mean (SD) | 66.0 (35.0) | 74.1 (34.5) | 0.026  (U) |
| Min-Max | 1 - 112 | 1 - 112 |  |
| *Days without treatment* | *534* | *358* | *NA* |
| **Length of treatment (days)** |  |  |  |
| No. | 171 | 174 | 0.023 (U) |
| Mean (SD) | 77.6 (36.9) | 86.1 (35.1) |  |
| Min-Max | 5 - 112 | 3 - 112 |  |
| **Length of NPWT (days)** |  |  |  |
| No. (%) | 168 (98.2) | 18 (10.3) | NA |
| Mean (SD) | 30.1 (26.8) | 28.5 (21.1) |  |
| Min-Max | 1 - 112 | 2 - 82 |  |
| **Length of inpatient NPWT** |  |  |  |
| No. (%) | 133 (77.8) | 18 (10.3) |  |
| Mean (SD) | 13.4 (11.5) | 22.4 (20.1) | NA |
| Min-Max | 1 – 92 | 2 – 82 |  |
| **Length of outpatient NPWT (days)** |  |  |  |
| No. (%) | 112 (65.5) | 7 (4.0) | NA |
| Mean (SD) | 29.3 (26.3) | 15.6 (17.6) |  |
| Min-Max | 1 – 112 | 1 – 42 |  |
| **Length of SMWC (days)** |  |  |  |
| No. (%) | 154 (90.1) | 174 (100) | NA |
| Mean (SD) | 53.3 (34.3) | 83.2 (35.3) |  |
| Min-Max | 1 – 110 | 3 – 112 |  |
| **Length of inpatient SMWC (days)** |  |  |  |
| No. (%) | 93 (54.4) | 142 (81.6) |  |
| Mean (SD) | 12.6 (12.3) | 19.5 (20.7) | NA |
| Min-Max | 1 – 55 | 1 – 112 |  |
| **Length of outpatient SMWC (days)** |  |  |  |
| No. (%) | 144 (84.2) | 163 (93.7) |  |
| Mean (SD) | 50.0 (32,5) | 71.9 (34.1) | NA |
| Min-Max | 1 – 110 | 1 – 112 |  |
| **Study participants still in care at day 112, No. (%)** | 61 (35.7) | 80 (46.0) | 0.052 (Chi^2^) |
| Study participants still in inpatient care at day 112, No. (%) | 3 (4.9) | 5 (6.3) | 0.735  (Chi²) |
| Study participants still in outpatient care at day 112, No. (%) | 58 (95.1) | 75 (93.8) |  |
| Study participants without treatment at Day 112, No. (%) | 3 (4.9) | 3 (3.8) | NA |
| Study participants with NPWT at Day 112, No. (%) | 6 (9.8) | 1 (1.3) | NA |
| Study participants with SMWC at Day 112, No. (%) | 52 (85.2) | 76 (95.0) | NA |

# Table 16: Time until finally achieving 95% granulation of the study wound and treatment continuation after optimal preparation for further therapeutic measures within 16 weeks in the ITT population

| **Randomized treatments arms / statistical test** | **NPWT** | **SMWC** | **p value**  **(Test)** |
| --- | --- | --- | --- |
| Study participants in the ITT population, No. | 171 | 174 | NA |
| **Study participants without achieving 95% granulation of the study wound, No. (%)** | 86 (50.3) | 99 (56.9) | 0.219  (Chi²) |
| **Study participants achieving 95% granulation of the study wound, No. (%)** | 85 (49.7) | 75 (43.1) |  |
| **Time until achieving 95% granulation of the study wound within 16 weeks** |  |  |  |
| Mean (SD) | 44.3 (30.7) | 56.0 (37.4) |  |
| [95% CI] | [36.8 - 51.8] | [46.5 - 65.5] | 0.003  (U) |
| Min-Max | 0 – 112 | 0 - 115 |  |
| **Study participants without data after 95% granulation of the study wound, No. (%)** | 15 of 85 (17.6) | 24 of 75 (32.0) | NA |
| **Study participants with data after 95% granulation of the study wound, No. (%)** | 70 of 85 (82.4) | 51 of 75 (68.0) | NA |
| **Study participants with SMWC after 95% granulation of the study wound, No. (%)** | 58 of 70 (82.9) | 44 of 51 (86.3) | NA |
| **Length of SMWC treatment after 95% granulation of the study wound** |  |  |  |
| Mean (SD) | 53.6 (31.4) | 60.8 (35.8) | 0.229 |
| Min-Max | 1 – 107 | 1 – 112 | (U) |
| Length of inpatient SMWC treatment after 95% granulation of the study wound |  |  |  |
| No. | 21 | 23 |  |
| Mean (SD) | 15.8 (13.8) | 12.5 (10.0) | 0.517 |
| Min-Max | 1 – 56 | 1 – 35 | (U) |
| Length of outpatient SMWC treatment after 95% granulation of the study wound |  |  |  |
| No. | 57 | 40 |  |
| Mean (SD) | 48.7 (30.9) | 60.1 (32.4) | 0.080 |
| Min-Max | 1 – 107 | 1 – 112 | (U) |

# Table 17: Hospital discharge of study participants with inpatient study start, outpatient treatment continuation and wound closures until the end of the maximum study treatment time of 16 weeks in the ITT population

| **Randomized treatments arms / statistical test** | **NPWT** | **SMWC** | **p value**  **(Test)** |
| --- | --- | --- | --- |
| Study participants in the ITT population, No. | 171 | 174 | NA |
| Study participants with outpatient study start, No. (%) | 44 (25.7) | 44 (25.3) | 0.925  (Chi²) |
| Study participants with study start in hospital, No. (%) | 127 (74.3) | 130 (74.7) |  |
| Study participants with study start in hospital and discharged within 16 weeks, No. (%) | 119 of 127 (93.7) | 120 of 130 (92.3) | 0.662  (Chi²) |
| Time until first discharge from hospital during the study treatment period of 42 days (days) |  |  |  |
| Mean (SD) | 13.8 (12.0) | 12.3 (13.1) | 0.071  (U) |
| Min-Max | 1 – 69 | 1 – 75 |  |
| Study participants with treatment continuation after hospital discharge, No. (%)  *For one study participants with NPWT no data were available after hospital discharge. | 109 of 119 (91.6) | 116 of 120 (96.7) | NA |
| Study participants with **NPWT after hospital discharge**, No. (%) | 59 of 109 (54.1) | 8 of 116 (6.9) | NA |
| Length of NPWT after hospital discharge (days) |  |  |  |
| Mean (SD) | 34.6 (27.5) | 27.5 (18.5) | NA |
| Min - Max | 2 - 107 | 2 - 55 |  |
| Study participants with **NPWT and SMWC after hospital discharge,** No. (%) | 53 (48.6) | 8 (6.9) | NA |
| Length of SMWC additionally to NPWT after hospital discharge (days) |  |  |  |
| Mean (SD) | 41.2 (29.3) | 68.8 (23.5) | NA |
| Min - Max | 0 - 100 | 30 - 100 |  |
| Study participants with NPWT after hospital discharge and hospital readmission, No. (%) | 31 (52.5) | 6 (75.0) | NA |
| Study participants with NPWT after hospital discharge and wound closure without counter evidence, No. (%) [95% CI] | 10 (16.9)  [7.4-26.5] | 0 (0) | NA |
| Time until wound closure without counter evidence for study participants with NPWT after hospital discharge |  |  |  |
| Mean (SE) | 72.5 (19.1) | NA | NA |
| [95% KI] | [58.8 - 86.2] |  |  |
| Study participants with **SMWC only after hospital discharge**, No. (%) | 50 of 109 (45.9) | 108 of 116 (93.1) | NA |
| Length of SMWC after hospital discharge (days) |  |  |  |
| Mean (SD) | 67.3 (34.0) | 77.9 (31.4) | NA |
| Min - Max | 3 - 109 | 4 - 110 |  |
| Study participants with SMWC only after hospital discharge and hospital readmission, No. (%) | 42 (40.8) | 32 (27.6) | NA |
| Study participants with SMWC only after hospital discharge and wound closure without counter evidence, No. (%) [95% CI] | 12 (24.0)  [12.2-35.8] | 18 (16.7)  [9.6-23.7] | NA |
| Time until wound closure for study participants with SMWC only after hospital discharge |  |  |  |
| Mean (SE) | 59.6 (30.2) | 81.7 (24.0) | NA |
| [95% CI] | [40.4-78.8] | [69.8-93.7] |  |
| Study participants with inpatient study start, hospital discharge and readmission, No. (%) | 44 of 127 (34.6) | 32 of 130 (24.6) | NA |
| Study participants with outpatient study start and hospital admission, No. (%) | 12 of 171 (7.0) | 14 of 174 (8.0) | NA |
| Study participants with hospital admission / readmission, No. (%) | 56 of 171 (32.7) | 46 of 174 (26.4) | NA |

# Table 18: Local wound treatment within the follow-up period in the ITT population

| **Randomized treatments arms / statistical test** | **NPWT** | **SMWC** |
| --- | --- | --- |
| Study participants in the ITT population, No. | 171 | 174 |
| Study participants with available data on local wound treatment during the follow-up period, No. | 114 | 127 |
| Study participants without local wound treatment during the follow-up period, No. (%) | 52 of 114 (45.6%) | 47 of 127 (37.0%) |
| Study participants with local wound treatment during the follow-up period, No. (%) | 62 of 114 (54.4) | 80 of 127 (63.0) |
| Study participants with data on type of local wound treatment, No. | 60 of 62 (96.8) | 80 of 80 (100) |
| Study participants with NPWT during the follow-up period, No. (%) | 2 of 60 (3.3) | 2 of 80 (2.5) |
| Study participants with SMWC during the follow-up period, No. (%) | 58 of 60 (96.7) | 78 of 80 (97.5) |
| Study participants with data on start and end of local wound treatment during the follow-up period, No. | 15 of 62 (24.1) | 26 of 80 (32.5) |
| Length of local wound treatment during the follow-up period (days) |  |  |
| Mean (SD) | 117.0 (57.2) | 119.5 (69.6) |
| Min - Max | 14 - 199 | 1 - 235 |
| Study participants with NPWT and data on start and end of local wound treatment during the follow-up period, No. | 1 | 1 |
| Length of NPWT during the follow-up period (days) |  |  |
| Mean (SD) | 158 (NA) | 20 (NA) |
| Min - Max | 158 - 158 | 20 - 20 |
| Study participants with SMWC and data on start and end of local wound therapy during the follow-up period, No. | 13 | 25 |
| Length of NPWT during the follow-up period (days) |  |  |
| Mean (SD) | 118.8 (57.8) | 123.4 (68.0) |
| Min - Max | 14 - 199 | 1 - 235 |

# Table 19: Post hoc analysis of wound closures without counter evidence within 16 weeks in the ITT population

| **Randomized treatments arms / statistical test** | **NPWT** | **SMWC** | **p value**  **(Test)** |
| --- | --- | --- | --- |
| Study participants in the ITT population, No. | 171 | 174 | NA |
| Study participants without wound closure without counter evidence within 16 weeks, No. (%) | 131 (76.6) | 142 (81.6) | 0.253  (Chi^2^) |
| Study participants with wound closure without counter evidence within 16 weeks, No. (%) 95%CI | 40 (23.4)  17.0 – 29.7 | 32 (18.4)  12.6 – 24.1 |  |
| Time until wound closure without counter evidence within 16 weeks, Median (SE) [95%CI] | 57.0 (6.3)  [44.6 – 69.4] | 80.0 (4.5)  [71.1 – 88.9] | 0.031  (Log-Rank) |

# Table 20: Dressing changes within 16 weeks in the ITT population

| **Randomized treatments arms / statistical test** | **NPWT** | **SMWC** | **p value**  **(test)** |
| --- | --- | --- | --- |
| Study participants in the ITT population, N | 171 | 174 | NA |
| Study participants without dressing changes, No. | 1 | 2 | NA |
| Study participants with dressing changes, No. | 170 | 172 | NA |
| Study participants with at least one missing record on the number of dressing changes between study visits, No. | 14 | 10 | NA |
| Study participants with at least one record on the number of dressing changes between study visits, No. | 169 | 172 | NA |
| Number of dressing changes per study participant |  |  |  |
| Mean (SD) | 30.2 (18.9) | 36.9 (21.9) | 0.004  (U) |
| Min – Max | 2 - 100 | 1 - 115 |  |
| Study participants with at least one missing personnel record for dressing changes between study visits, No. | 4 | 0 | NA |
| Study participants with at least one personnel record for dressing changes between study visits, No. | 170 | 172 | NA |
| Study participants with **outpatient nursing service** involvement in dressing changes between study visits, No. | 67 | 69 | NA |
| Dressing changes with outpatient nursing service involvement per study participant |  |  |  |
| Mean (SD) | 18.5 (14.7) | 22.1 (16.0) | NA |
| Min – Max | 2 - 81 | 1 - 65 |  |
| Study participants with **outpatient treatment facility** involvement in dressing changes between study visits, No. | 103 | 92 | NA |
| Dressing changes with outpatient treatment facility involvement per study participant |  |  |  |
| Mean (SD) | 13.2 (10.9) | 17.4 (11.8) | NA |
| Min – Max | 1 - 45 | 2 - 50 |  |
| Study participants with **inpatient treatment facility** involvement in dressing changes between study visits, No. | 128 | 130 | NA |
| Dressing changes with inpatient treatment facility involvement per study participant |  |  |  |
| Mean (SD) | 9.6 (8.6) | 11.7 (13.8) | NA |
| Min – Max | 1 - 39 | 1 - 82 |  |
| Study participants with other personnel involved in dressing changes between study visits, No. | 27 | 27 | NA |
| Dressing changes with other personnel involved per study participant |  |  |  |
| Mean (SD) | 14.3 (17.7) | 20.5 (16.6) | NA |
| Min – Max | 2 - 89 | 3 - 61 |  |

# Table 21: Exemplary record of time and personnel spent on dressing changes at the current study visit within the study treatment period of 16 weeks in the ITT population.

| **Randomized treatments arms / statistical test** | **NPWT** | **SMWC** | **p value**  **(test)** |
| --- | --- | --- | --- |
| Study participants in the ITT population, No. | 171 | 174 | NA |
| Study participants with at least one documented dressing change during a study visit, No. | 171 | 173 | NA |
| **Study participants with at least one time record for dressing changes during a study visit, No.** | **171** | **173** | **NA** |
| Study participants with at least one missing time record for dressing changes during a study visit, No. | 3 | 6 | NA |
| Time required per dressing change |  |  |  |
| Mean (SD) | 20.5 (12.0) | 17.5 (8.2) | <0.0001  (U) |
| Min - Max | 1 - 150 | 3 - 60 |  |
| **Study participants with at least one personnel record for dressing changes during a study visit, No.** | **171** | **172** | **NA** |
| Study participants with at least one missing personnel record for dressing changes during a study visit, No. | 3 | 12 | NA |
| Study participants with **nursing assistance** contacts for dressing changes within the study visit, No. (%) | 18 of 171 (10.5) | 17 of 172  (9.9) | NA |
| Study participants with **nurse** contacts for dressing changes within the study visit, No. (%) | 141 of 171 (82.5) | 143 of 172 (83.1) | NA |
| Study participants with **assistant physician** contacts for dressing changes within the study visit, No. (%) | 30 of 171 (17.5) | 35 of 172 (20.3) | NA |
| Study participants with **specialist physician** contacts for dressing changes within the study visit, No. (%) | 94 of 171 (55.0) | 96 of 172 (55.8) | NA |
| Study participants with **other personnel** contacts for dressing changes within the study visit, No. (%) | 30 of 171 (17.5) | 30 of 172 (17.4) | NA |

# Table 22: Use of NPWT wound dressings and systems within the study treatment period of 16 weeks in the ITT population

| **Randomized treatments arms** | **NPWT** | **SMWC** |
| --- | --- | --- |
| Study participants in the ITT population, No. | 171 | 174 |
| **Study participants with data on the use of NPWT wound dressings, No.** | **158** | **18** |
| **Study participants with data on the use of KCI NPWT wound dressings, No.** | **142** | **18** |
| Study participants with KCI - Granufoam^®^ black, No. | 120 | 15 |
| Study participants with KCI - White Foam^®^, No. | 13 | 0 |
| Study participants with KCI - Granufoam^®^ black and KCI - White Foam^®^, No. | 6 | 1 |
| Study participants with KCI - Granufoam^®^ black and KCI - Silver^®^, No. | 2 | 1 |
| Study participants with KCI - Silver^®^ and KCI - White Foam^®^, No. | 1 | 1 |
| **Study participants with data on the use of S&N NPWT wound dressings, No.** | **15** | **0** |
| Study participants with S&N - wound foam (Renassys^TM^ –F/P) | 15 | 0 |
| **Study participants with data on the use of KCI and S&N NPWT wound dressings, No.** | 1 | 0 |
| Study participants with KCI - White Foam^®^ and S&N - wound foam (Renassys^TM^ –F/P) | 1 | 0 |
| **Study participants with data on the use of NPWT systems, No.** | **158** | **17** |
| **Study participants with data on the use of KCI NPWT systems, No.** | **142** | **17** |
| Study participants with KCI-V.A.C. Freedom^®^, No. | 8 | 1 |
| Study participants with KCI-Acti V.A.C.^®^, No. | 116 | 14 |
| Study participants with KCI-V.A.C. Freedom^®^ and KCI-Acti V.A.C.^®^, No. | 4 | 0 |
| Study participants with KCI-V.A.C. Freedom^®^ and KCI-INFO V.A.C.^®^, No. | 2 | 0 |
| Study participants with KCI-Acti V.A.C.^®^ and KCI-INFO V.A.C.^®^, No. | 9 | 1 |
| **Study participants with data on the use of S&N NPWT systems, No.** | **15** | **0** |
| Study participants with S&N-RenasysTM GO | 15 | 0 |
| **Study participants with data on the use of KCI and S&N NPWT systems, No.** |  |  |
| Study participants with KCI-V.A.C. Freedom^®^ and S&N-RenasysTM GO | 1 | 0 |

# Table 23: Use of SMWC dressings within the study treatment period of 16 weeks in the ITT population

| **Randomized treatments arms** | **NPWT** | **SMWC** |
| --- | --- | --- |
| Study participants in the ITT population, No. | 171 | 174 |
| Study participants at least once treated with SMWC, No. (%) | 154 (90.1) | 174 (100) |
| Study participants with at least one documentation of a wound dressing (wound filler or wound cover*) within the study treatment period, No. (%) | 154 (90.1) | 174 (100) |
| **Study participants with at least one documentation of a wound filler, No.** | 134 | 170 |
| Hydrogel and hydrogel coated hydrophobic dressings, No. (%) | 47 of 134 (35.1) | 80 of 170 (47.1) |
| Hydrogel, No. | 44 | 69 |
| Hydrogel coated hydrophobic dressings, No. | 3 | 11 |
| Alginate with and without silver, No. (%) | 60 of 134 (44.8) | 71 of 170 (41.8) |
| Alginate, No. | 45 | 52 |
| Alginate with silver, No. | 15 | 19 |
| Hydrofiber with and without silver, No. (%) | 37 of 134 (27.6) | 38 of 170 (22.4) |
| Hydrofiber, N | 26 | 30 |
| Hydrofiber with silver, N | 11 | 8 |
| Foams with and without silver or antiseptics, No. (%) | 34 of 134 (25.4) | 62 of 170 (36.5) |
| Polyurethane (PU) foam, tamponade, cavity, No. | 32 | 57 |
| Polyurethane (PU) foam, tamponade, cavity with silver, No. | 2 | 4 |
| Polyurethane (PU) foam, tamponade, cavity with antiseptics, No. | 0 | 1 |
| Collagen, No. (%) | 5 of 134 (3.7) | 3 of 170 (1.8) |
| Hyaluronic acid, No. (%) | 1 of 134 (0.7) | 0 of 170 (0) |
| Hydrophobic materials, No. (%) | 38 of 134 (28.4) | 45 of 170 (26.5) |
| Silver dressing materials with activated carbon, No. (%) | 1 of 134 (0.7) | 8 of 170 (4.7) |
| Silver dressing materials without activated carbon, No. (%) | 7 of 134 (5.2) | 9 of 170 (5.3) |
| Gauze and fleece compresses, tamponades partially soaked with antiseptics, No. (%) | 10 of 134 (7.5) | 12 of 170 (7.1) |
| Gauze partially soaked with antiseptic or hemostatic agent, No. (%) | 2 of 134 (1.5) | 1 of 170 (0.6) |
| Other indications which, by definition, are not wound fillers or cannot be clearly assigned to a category, No. (%) | 1 of 134 (0.7) | 4 of 170 (2.4) |
| Maggots / Maggot therapy, No. | 1 | 1 |
| Suprasorb, No. | 0 | 2 |
| Biatain. No. | 0 | 1 |
| Other wound filler without specification, No. (%) | 7 of 134 (5.2) | 7 of 170 (4.1) |
| **Study participants with at least one documentation of a wound cover, No.** | 153 | 173 |
| Gauze compresses, No. (%) | 97 of 153 (63.4) | 115 of 173 (66.5) |
| Nonwoven compresses, No. (%) | 15 of 153 (9.8) | 20 of 173 (11.6) |
| Absorbent compresses with cellulose core partially activated with Ringer's solution, No. (%) | 31 of 153 (20.3) | 53 of 173 (30.6) |
| Absorbent compresses with cellulose core, No. | 30 | 50 |
| Absorbent compresses with cellulose core activated with Ringer's solution, No. | 1 | 3 |
| Superabsorbent dressings, No. (%) | 7 of 153 (4.6) | 14 of 173 (8.1) |
| Films sterile and non-sterile, No. (%) | 3 of 153 (2.0) | 2 of 173 (1.2) |
| Films sterile, No. | 3 | 0 |
| Films non-sterile, No. | 0 | 2 |
| Hydrocolloids, No. (%) | 6 of 153 (3.9) | 3 of 173 (1.7) |
| Hydrofiber with and without silver, No. (%) | 2 of 153 (1.3) | 2 of 173 (1.2) |
| Adhesive hydrofiber, No. | 0 | 0 |
| Non-adhesive hydrofiber, No. | 1 | 1 |
| Non-adhesive hydrofiber with silver, No. | 1 | 1 |
| Foams with and without ibuprofen or silver, No. (%) | 86 of 153 (56.2) | 120 of 173 (69.4) |
| PU foam adhesive, No. | 12 | 25 |
| PU foam soft adhesive, No. | 39 | 43 |
| PU foam non-adhesive, No. | 35 | 47 |
| PU foam non-adhesive with Ibuprofen, No. | 0 | 2 |
| PU foam non-adhesive with silver, No. | 0 | 3 |
| Odor-reducing dressing materials with activated carbon, No. (%) | 0 of 153 (0) | 0 of 173 (0) |
| Hydrophobic materials with and without silver or honey | 29 of 153 (19.0) | 28 of 173 (16.2) |
| Hydrophobic materials, No. | 26 | 25 |
| Hydrophobic materials with silver, No. | 3 | 2 |
| Hydrophobic materials with honey, No. | 0 | 1 |
| Silver dressings with activated charcoal, No. (%) | 3 of 153 (2.0) | 6 of 173 (3.5) |
| Silver dressings without activated charcoal, No. (%) | 5 of 153 (3.3) | 2 of 173 (1.2) |
| Other material without direct effect on the wound / additives, No. (%) | 45 of 153 (29.4) | 57 of 173 (32.9) |
| Cushioning material and bandages, fixatives, compression bandages and abdominal sheets, No. | 21 | 32 |
| Steri-strips, No. | 2 | 2 |
| Plaster, No. | 4 | 11 |
| Additive of antiseptics without specification of the dressing, No. | 17 | 11 |
| Protection, No.: | 1 | 1 |
| *Mepilex Border for skin protection, No.* | 0 | 1 |
| *For protection, No.* | 1 | 0 |
| Other wound covers without specification, No. (%) | 9 of 153 (5.9) | 10 of 173 (5.8) |

*Multiple answers were possible

# Table 24: Wound cleansing and decontamination within the study treatment period of 16 weeks in the ITT population

| **Randomized treatments arms / statistical test** | **NPWT** | **SMWC** | **p value**  **(Test)** |
| --- | --- | --- | --- |
| Study participants in the ITT population, N | 171 | 174 | NA |
| Study participants without wound cleansing or decontamination, No. (%) | 0 of 171 (0) | 0 of 174 (0) | NA |
| Study participants with wound cleansing or decontamination, No. (%) | 171 of 171 (100) | 168 of 174 (100) | NA |
| Number of wound cleansings and decontaminations | 3922 | 4558 | NA |
| Wound cleansings and decontaminations per study participant  Mean (SD)  Min - Max | 22.9 (16.2)  1 - 99 | 26.2 (19.0)  1 - 91 | (U) |
| **Type of wound cleansing and decontamination*** |  |  |  |
| Study participants with debridement to intact anatomic structures, No. (%) | 74 of 171 (43.3) | 80 of 174 (46,0) | NA |
| Study participants with mechanical wound cleansing, No. (%) | 110 of 171 (64,3) | 114 of 174 (65.5) | NA |
| Study participants with wound irrigation, No. (%) | 111 of 171 (64.9) | 105 of 174 (60.3) | NA |
| Study participants with wrappings and long-term wet periods, No. (%) | 6 of 171 (3.5) | 5 of 174 (2.9) | NA |
| Study participants with use of antiseptics, No. (%) | 38 of 171 (22.2) | 35 of 174 (20.1) | NA |
| Study participants with ultrasonic cleaning, No. (%) | 0 of 171 (0) | 2 of 174 (1.1) | NA |
| Study participants using moisturizing materials, No. (%) | 5 of 171 (2.9) | 2 of 174 (1.1) | NA |
| Study participants with enzymatic wound cleansing, No. (%) | 1 of 171 (0.6) | 2 of 174 (1.1) | NA |
| Study participants with wound cleaning by fly maggots, No. (%) | 5 of 171 (2.9) | 6 of 174 (3.4) | NA |
| Study participants with osmotic wound cleansing, No. (%) | 0 of 171 (0) | 0 of 174 (0) | NA |
| Study participants with other wound cleansing and decontamination methods, No. (%) | 10 of 171 (5.8) | 6 of 174 (3.4) | NA |

*Multiple answers were possible

# Table 25: Surgical debridement within the study treatment period of 16 weeks in the ITT population

| **Randomized treatments arms /statistical test** | **NPWT** | **SMWC** | **p value**  **(Test)** |
| --- | --- | --- | --- |
| Study participants in the ITT population, No. | 171 | 174 | NA |
| Study participants without debridement, No. (%) | 128 (74.9) | 123 (70.7) | 0.385  (Chi^2^) |
| Study participants with debridement, No. (%) | 43 (25.1) | 51 (29.3%) |  |
| Number of debridements | 174 | 215 | NA |
| Debridements per study participant |  |  |  |
| Mean (SD) | 4.1 (4.5) | 4.4 (5.4) | 0.833  (U) |
| Min - Max | 1 – 21 | 1 – 25 |  |
| Study participants with available total duration of debridements, No. | 39 | 39 | NA |
| Number of debridements with time expenditure | 96 | 107 | NA |
| *Study participants with at least one missing indication of debridement duration, No.* | 9 | 14 | NA |
| *Missing information on the duration of debridement, No.* | 16 | 27 | NA |
| Time spent per debridement |  |  |  |
| Mean (SD) | 21.9 (26.2) | 30.7 (66.9) | 0.419  (U) |
| Min - Max | 0 - 180 | 1 - 490 |  |
| Time spent for debridements per study participant |  |  |  |
| Mean (SD) | 29.9 (24.5) | 35.5 (40.4) | (U) |
| Min - Max | 0 – 90 | 1 – 230 |  |

# Table 26: Wound margin and wound environment protection within the study treatment period of 16 weeks in the ITT population

| **Randomized treatments arms / statistical test** | **UWT** | **SWT** | **p value**  **(test)** |
| --- | --- | --- | --- |
| Study participants in the ITT population, No. | 171 | 174 | NA |
| Study participants with wound margin and wound environment protection measures implemented, No. (%) | 137 (80.1) | 143 (82.2%) | 0.624  (Chi^2^) |
| **Type of protective measures*:** |  |  |  |
| Study participants with hyperkeratosis ablation, No. (%) | 66 of 137 (48.2) | 85 of 143 (59.4) | NA |
| Study participants with skin care measures, No. (%) | 109 of 137 (79.6) | 131 of 143 (91.6) | NA |
| Study participants with skin protection measures, No. (%) | 103 of 137 (75.2) | 69 of 143 (48.3) | NA |
| Study participants with mycosis therapy, No. (%) | 2 of 137 (1.5) | 0 of 143 (0) | NA |
| Study participants with measures against contact allergy, No. (%) | 3 of 137 (2.2) | 0 of 143 (0) | NA |
| Study participants with other wound margin and wound environment protection measures, No. (%) | 8 of 137 (5.8) | 7 of 143 (4.9) | NA |

*Multiple answers were possible

# Table 27: Pressure relief within the study period of 16 weeks in the ITT population

| **Randomized treatments arms** | **NPWT** | **SMWC** | **p value**  **(test)** |
| --- | --- | --- | --- |
| Study participants in the ITT population, No. | 171 | 174 |  |
| Study participants without data on pressure relief, No. (%) | 2 (1.2%) | 4 (2.3%) | 0.873  (Chi^2^) |
| Study participants with pressure relief measures, No. (%) | 169 (98.3%) | 170 (97.7%) |  |
| Study participants with pressure relief measures, but at least once without indication of type of protective measure, No. | 9 | 6 | NA |
| Study participants with pressure relief measures, but at least once without indication of type of protective measure but with indication if pressure relief was ensured, No. | 9 | 6 | NA |
| Study participants with pressure relief measures and at least one indication of the type of protective measure, No. (%) | 167 (98.8) | 170 (100) | NA |
| **Study participants with the respective type of pressure relief measure:** |  |  |  |
| Protective footwear: relief shoes, No. (%) | 116 of 167 (69.5) | 115 of 170 (67.6) | NA |
| Protective footwear: interim shoes, No. (%) | 20 of 167 (12.0) | 19 of 170 (11.2) | NA |
| Footwear with diabetes-adapted footbed, No. (%) | 22 of 167 (13.2) | 27 of 170 (15.9) | NA |
| Orthopedic fittings, No. (%) | 12 of 167 (7.2) | 11 of 170 (6.5) | NA |
| Total-Contact cast, No. (%) | 6 of 167 (3.6) | 7 of 170 (4.1) | NA |
| Scotch cast boots, No. (%) | 0 of 167 (0) | 0 of 170 (0) | NA |
| Ready-made or custom-made orthoses, No. (%) | 23 of 167 (13.8) | 32 of 170 (18.8) | NA |
| Walking aids, No. (%) | 25 of 167 (15.0) | 32 of 170 (18.8) | NA |
| Wheel chair, No. (%) | 40 of 167 (24.0) | 38 of 170 (22.4) | NA |
| Initial bed rest, No. (%) | 33 of 167 (19.8) | 35 of 170 (20.6) | NA |
| Other: Positioning or positioning material, No. (%) | 2 of 167 (1.2) | 2 of 170 (1.2) | NA |
| Other: description of non-established methods, No. (%): | 3 of 167 (1.8) | 1 of 170 (0.6) | NA |
| Cut out Crocs, No. | 1 | 0 | NA |
| Bandage, No. | 1 | 0 | NA |
| Cellona cushion, No. | 0 | 1 | NA |
| **Ensuring pressure relief per study participant:** |  |  |  |
| Complete, No. (%) | 72 of 169 (42.6) | 75 of 170 (44.1) | NA |
| Partial, No. (%) | 53 of 169 (31.4) | 56 of 170 (32.9) | NA |
| Not at all, No. (%) | 0 of 169 (0) | 0 of 170 (0) | NA |
| Complete & partial, No. (%) | 40 of 169 (23.7) | 38 of 170 (22.4) | NA |
| Complete & not at all, No. (%) | 1 of 169 (0.6) | 0 of 170 (0) | NA |
| Complete & partial & not at all, No. (%) | 3 of 169 (1.8) | 0 of 170 (0) | NA |
| Complete & not at all, No. (%) | 0 of 169 (0) | 1 of 170 (0.6) | NA |
| Partial & not at all, No. (%) | 72 of 169 (42.6) | 75 of 170 (44.1) | NA |

*Multiple answers were possible

# Table 28: Vascular occlusion locations within the study treatment period of 16 weeks in the ITT population

| **Randomized treatments arms** | **NPWT** | **SMWC** |
| --- | --- | --- |
| Study participants in the ITT population, No. | 171 | 174 |
| Study participants with central occlusion, No. | 0 | 1 |
| Study participants with inguinal occlusion, No. | 1 | 0 |
| Study participants with occlusion combination: femoral, popliteal, crural, No. | 4 | 1 |
| Study participants with occlusion combination: femoral, popliteal, pedal, No. | 0 | 1 |
| Study participants with occlusion combination: femoral, popliteal, No. | 5 | 5 |
| Study participants with occlusion combination: femoral, crural, No. | 6 | 6 |
| Study participants with occlusion combination: femoral, pedal, No. | 0 | 1 |
| Study participants with femoral occlusion, No. | 3 | 2 |
| Study participants with occlusion combination: popliteal, crural, No. | 0 | 1 |
| Study participants with popliteal occlusion, No. | 1 | 1 |
| Study participants with occlusion combination: crural, pedal, No. | 1 | 0 |
| Study participants with crural occlusion, No. | 3 | 5 |

# Table 29: Revascularization within the study treatment period of 16 weeks in the ITT population

| **Randomized treatments arms** | **NPWT** | **SMWC** | **p value**  **(Test)** |
| --- | --- | --- | --- |
| Study participants in the ITT population, No. | 171 | 174 | NA |
| Study participants with at least one revascularization, No. (%) | 24 (14.0%) | 22 (12.6%) | 0.704  (Chi^2^) |
| Study participants with one revascularization, No. | 19 | 19 | NA |
| Study participants with two revascularizations, No. | 4 | 3 | NA |
| Study participants with three revascularizations, No. | 0 | 0 | NA |
| Study participants with four revascularizations, No. | 1 | 0 | NA |
| Number of revascularizations | 31 | 25 | NA |
| Revascularizations per study participant |  |  |  |
| Mean (SD)  Min - Max | 0.2 (0.5)  0 - 4 | 0.1 (0.4)  0 - 2 | 0.677  (U) |
| Study participants with at least one revascularization procedure performed with effect on the study wound, No. | 23 | 22 | NA |
| Study participants with at least one revascularization procedure performed without effect on the study wound, No. | 0 | 0 | NA |
| **Type of revascularization:** |  |  |  |
| Study participants with multilevel reconstruction, No. | 1 | 1 | NA |
| Study participants with thrombarterioectomy (TEA) and patch plasty, No. | 1 | 1 | NA |
| Study participants with polytetrafluoroethylene (PTFE) bypass graft, No. | 2 | 0 | NA |
| Study participants with venous bypass, No. | 2 | 3 | NA |
| Study participants with percutaneous transluminal angioplasty (PTA), No. | 7 | 9 | NA |
| Study participants with the sequence: PTA & stent, venous bypass, PTA & stent, PTA & stent, No. | 1 | 0 | NA |
| Study participants with the sequence: PTA & Stent, PTA, No. | 0 | 1 | NA |
| Study participants with the sequence: PTA & Stent, No. | 2 | 3 | NA |
| Study participants with the sequence: PTA, venous bypass, No. | 1 | 0 | NA |
| Study participants with the sequence: PTA, PTA, No. | 3 | 2 | NA |
| Study participants with other techniques, No. | 3 | 2 | NA |
| **Revascularization result:** |  |  | NA |
| Study participants with sufficient revascularization result, No. | 22* | 21 | NA |
| Study participants with insufficient revascularization result, No. | 1 | 0 | NA |
| Study participants with sufficient revascularization result and non-assessable result, No. | 2* | 1 | NA |

*Multiple answers

# Table 30: Amputations within the study treatment period of 16 weeks in the ITT population

| **Randomized treatments arms** | **NPWT** | **SMWC** | **p value**  **(test)** |
| --- | --- | --- | --- |
| Study participants in the ITT population, No. | 171 | 174 | NA |
| Study participants without amputation, No. (%) | 137 (80.1%) | 138 (79.3%) | 0.934  (Chi^2^) |
| Study participants with amputation, No. (%) | 36 (21.1%) | 36 (20.7%) |  |
| Study participants with data on number of amputations, No. | 36 | 36 | NA |
| Number of amputations | 45 | 57 | NA |
| Amputations per study participant |  |  |  |
| Mean (SD) | 1.3 (0.4) | 1.6 (1.0) | 0.313  (U) |
| Min - Max | 1.0 – 2.0 | 1.0 – 5.0 |  |
| Study participants with data on time spent for amputations, No. | 33 | 30 | NA |
| Study participants with at least one missing data on time spent for amputations, No. | 3 | 8 | NA |
| Number of missing data for time spent for amputations | 3 | 8 | NA |
| Time spent for amputations per study participant |  |  |  |
| Mean (SD) | 33.1 (24.2) | 46.1 (34.6) | 0.252  (U) |
| Min - Max | 5 - 130 | 15 - 140 |  |

# Table 31: Defect coverage within the study treatment period of 16 weeks in the ITT population

| **Defektdeckung** | **NPWT** | **SMWC** | **p value**  **(test)** |
| --- | --- | --- | --- |
| Study participants in the ITT population, No. | 171 | 174 | NA |
| Study participants without defect coverage, No. (%) | 144 (84.2) | 167 (96.0) | **0.0002**  **(Chi^2^)** |
| **Study participants with defect coverage, No. (%)** | **27 (15.8)** | **7 (4.0)** |  |
| Study participants with one defect coverage, No. | 19 | 3 | NA |
| Study participants with two defect coverages, No. | 7 | 3 | NA |
| Study participants with three defect coverages, No. | 1 | 1 | NA |
| Study participants with at least one missing indication of the number of defect coverages, No. | 0 | 0 | NA |
| Study participants with data on the number of defect coverages, No. | 27 | 7 | NA |
| Number of defect coverages | 34 | 11 | NA |
| Defect coverages per study participant |  |  |  |
| Mean (SD) | 1.3 (0.4) | 1.6 (0.5) | 0.121  (U) |
| Min – Max | 1 - 2 | 1 - 2 |  |
| Study participants with at least one missing indication of the duration of defect coverages, No. | 2 | 1 | NA |
| Number of missing data of the duration of the defect coverage | 2 | 1 | NA |
| Study participants with data on the duration of defect coverage, No. | 27 | 6 | NA |
| Duration of defect coverage per study participant |  |  |  |
| Mean (SD) | 37.6 (23.2) | 54.2 (23.2) | 0.224 |
| Min – Max | 10 – 120 | 30 – 120 | (U) |
| Number of defect coverages with indication of duration | 32 | 9 | NA |
| Duration per defect coverage |  |  |  |
| Mean (SD) | 31.7 (12.9) | 36.1 (13.6) | 0.527  (U) |
| Min – Max | 10 – 60 | 25 – 60 |  |
| Study participants with at least one missing indication of the type of defect coverage, No. | 1 | 1 | NA |
| Number of missing data of the type of defect coverage | 2 | 1 | NA |
| Study participants with data on the of type of defect coverage, No. | 26 | 6 | NA |
| **Study participants with skin graft, No. (%)** | **25 of 26 (96.2)** | **5 of 6 (83.3)** | **NA** |
| Number of skin grafts | 31 | 8 | NA |
| Skin grafts per study participant |  |  | NA |
| Mean (SD) | 1.2 (0.4) | 1.6 (0.5) |  |
| Min – Max | 1 – 2 | 1 – 2 |  |
| Study participants with data on the duration of skin grafts, No. | 25 | 4 | NA |
| Duration of skin grafts per study participant |  |  | NA |
| Mean (SD) | 38.2 (24,1) | 66.3 (38.2) |  |
| Min – Max | 10 – 120 | 30 – 120 |  |
| Number of skin grafts with indication of duration | 30 | 7 | NA |
| Duration per skin graft (Minutes) |  |  |  |
| Mean (SD) | 31.8 (13.4) | 37.9 (15.2) | NA |
| Min – Max | 10 – 60 | 25 - 60 |  |
| **Study participants with local flaps** | **1 of 26 (3.8)** | **1 of 6 (16.7)** | NA |
| Number of local flaps | 1 | 1 | NA |
| Duration per local flap (minutes) | 30 | 30 | NA |
